# An update on the efficacy of anti-inflammatory agents for patients with schizophrenia: a meta-analysis

**DOI:** 10.1017/S0033291719001995

**Published:** 2019-08-23

**Authors:** N. Çakici, N. J. M. van Beveren, G. Judge-Hundal, M. M. Koola, I. E. C. Sommer

**Affiliations:** 1 Department of Psychiatry and Amsterdam Neuroscience, Academic Medical Center, Meibergdreef 9, 1105 AZ Amsterdam, the Netherlands; 2 Antes Center for Mental Health Care, Albrandswaardsedijk 74, 3172 AA, Poortugaal, the Netherlands; 3 Department of Psychiatry, Erasmus Medical Center, Doctor Molewaterplein 40, 3015 GD Rotterdam, the Netherlands; 4 Department of Neuroscience, Erasmus Medical Center, Doctor Molewaterplein 40, 3015 GD Rotterdam, the Netherlands; 5 Department of Psychiatry and Biomedical Sciences of Cells and Systems, University Medical Center Groningen, Deusinglaan 2, 9713AW Groningen, the Netherlands; 6 Department of Psychiatry and Behavioral Sciences, George Washington University School of Medicine and Health Sciences, 2300I St NW, Washington, DC 20052, USA

**Keywords:** Add-on antipsychotic therapy, estrogens, fatty acids, minocycline, N-acetylcysteine

## Abstract

**Background:**

Accumulating evidence shows that a propensity towards a pro-inflammatory status in the brain plays an important role in schizophrenia. Anti-inflammatory drugs might compensate this propensity. This study provides an update regarding the efficacy of agents with some anti-inflammatory actions for schizophrenia symptoms tested in randomized controlled trials (RCTs).

**Methods:**

PubMed, Embase, the National Institutes of Health website (http://www.clinicaltrials.gov), and the Cochrane Database of Systematic Reviews were systematically searched for RCTs that investigated clinical outcomes.

**Results:**

Our search yielded 56 studies that provided information on the efficacy of the following components on symptom severity: aspirin, bexarotene, celecoxib, davunetide, dextromethorphan, estrogens, fatty acids, melatonin, minocycline, N-acetylcysteine (NAC), pioglitazone, piracetam, pregnenolone, statins, varenicline, and withania somnifera extract. The results of aspirin [mean weighted effect size (ES): 0.30; *n* = 270; 95% CI (CI) 0.06–0.54], estrogens (ES: 0.78; *n* = 723; CI 0.36–1.19), minocycline (ES: 0.40; *n* = 946; CI 0.11–0.68), and NAC (ES: 1.00; *n* = 442; CI 0.60–1.41) were significant in meta-analysis of at least two studies. Subgroup analysis yielded larger positive effects for first-episode psychosis (FEP) or early-phase schizophrenia studies. Bexarotene, celecoxib, davunetide, dextromethorphan, fatty acids, pregnenolone, statins, and varenicline showed no significant effect.

**Conclusions:**

Some, but not all agents with anti-inflammatory properties showed efficacy. Effective agents were aspirin, estrogens, minocycline, and NAC. We observed greater beneficial results on symptom severity in FEP or early-phase schizophrenia.

## Introduction

The pathophysiology of schizophrenia is still not completely understood, but there is accumulating evidence that dysregulations in components of the immune system are fundamentally linked to the disease. While genetic associations show that people with schizophrenia on average have an immune system subtly more prone to activation, as expressed e.g. in major histocompatibility complex molecules (Debnath *et al*., 2013; Mokhtari and Lachman, 2016), its enhancers (Takao *et al*., 2013), and complement factor 4 (Sekar *et al*., 2016), environmental circumstances that naturally activate the immune system such as prenatal infection, trauma, and stress, may put components of the immune system (i.e. microglia) in an altered state of activity (Brown and Derkits, 2010; Fineberg and Ellman, 2013). Under such circumstances, microglia and other glia may reduce their neurotrophic function and produce less growth factors such as brain-derived neurotrophic factor (BDNF), leading to decreased proliferation of neurons, resulting in reduced connectivity and, finally, brain tissue degradation. In addition, pruning may be increased by opsonization of synaptic buds with activated complement (Nimgaonkar *et al*., 2017; Presumey *et al*., 2017). Glutamatergic and dopaminergic neurotransmissions are particularly vulnerable for an increased activation of microglia, which can induce or exacerbate positive, negative, and cognitive symptoms of schizophrenia (Muller and Schwarz, 2006; Muller and Dursun, 2011).

Over the years, many studies have presented evidence to support this theory. A schizophrenia genome-wide association study found associations between schizophrenia and certain genes that are involved in immune processes (Schizophrenia Working Group of the Psychiatric Genomics, 2014). Peripheral blood markers, such as BDNF, interleukin (IL)-10, and C-reactive protein (CRP), are associated with cognitive decline in schizophrenia (Liu *et al*., 2018; Man *et al*., 2018; Misiak *et al*., 2018). Interestingly, a recent study identified macrophages on the brain side of the endothelial wall in a subgroup of patients with schizophrenia but not in controls, demonstrating an influx of peripheral immune cells (Cai *et al*., 2018).

The immune hypothesis readily suggests a possible treatment for those patients with schizophrenia in which the underlying pathophysiology is related to a subtle increase in the activation of microglia. Many medications can decrease the production of pro-inflammatory factors; however, it is not certain whether these agents can induce microglia, astrocytes, and other cells to resume their normal neurotrophic functions (Chew *et al*., 2013; Sommer *et al*., 2014). For one frequently used anti-inflammatory drug, minocycline, Sellgren *et al*. showed that this drug was indeed able to reduce microglia engulfment of complement opsonized synapses in a stem cell model derived from patients (Sellgren *et al*., 2019). This finding suggests that at least minocycline, but perhaps also other anti-inflammatory drugs, can correct one of the basic mechanisms underlying schizophrenia. Yet, components that work *in vitro* do not always work *in vivo*.

In a previous meta-analysis on augmentation with anti-inflammatory medications, we showed beneficial results of aspirin, estrogens, and N-acetylcysteine (NAC) on symptom improvement in patients with schizophrenia (Sommer *et al*., 2014), though based on very few studies. However, since the publication of our previous meta-analysis, a substantial number of additional studies have investigated the same and other agents with potential anti-inflammatory properties, which could reinstate the balance between synaptogenesis and pruning in schizophrenia and possibly improve symptoms. We have listed in Table 1 treatments with known anti-inflammatory actions, how well the blood-brain-barrier (BBB) can be crossed, and their actions in the brain. This summary is incomplete, as many nutritional and herbal components also possess anti-inflammatory aspects. Additionally, many psychotropic agents such as antipsychotics, selective serotonin reuptake inhibitors, lithium, and valproate acid also have some anti-inflammatory actions. As shown in Table 1, most anti-inflammatory components have many functions, and their anti-inflammatory actions are just one of them and often not the most important one. Some of these agents have been given to patients with schizophrenia in an attempt to normalize the brain's immune system and to eventually reduce symptoms. Here, we quantitatively summarize all available evidence of drugs with some anti-inflammatory aspects studied in patients with schizophrenia in a double-blind randomized design.

**Table 1.** Main types of medication with anti-inflammatory actions

Anti-inflammatory components	Crosses BBB	Actions in the brain	References^a^
Antipsychotics	++	Dopamine receptor blockade (D2), ↑BDNF	(Benros *et al*., 2012; Wakade *et al.*, 2002)
Aspirin	+/−	PG↓, TNF-*α*↓, COX-1↓, COX-2↓	(Roth and Majerus, 1975; Vane *et al.*, 1998)
Bexarotene	++	Complement system↓ (indirectly)	(Tousi, 2015; Yin *et al*., 2019)
Celecoxib	++	COX-2↓, PG↓	(Simon, 1999)
Corticosteroids	+/−	Inhibition of many steps in innate and specific immune response	(Liu *et al*., 2013)
Cytostatics	+/−	Diverse, e.g. for MTX: TNF-*α*↓	(Chan and Cronstein, 2010; Aletaha and Smolen, 2018)
Davunetide	++	TNF-*α*↓	(Quintana et al., 2006)
Dextromethorphan	++	Microglia inhibition	(Zhang *et al*., 2004)
Estrogens	++	IL-1β↓, IL-6↓, TNF-*α*↓, NF-κβ↓, NO↓	(Medina-Estrada *et al.*, 2018)
Fatty acids	++	Zinc↓, TNF-*α*↓, COX-2↓, IL-1↓	(Calder, 2012; Sadli *et al.*, 2012)
Leptin	++	Pro- and anti-inflammatory effects (e.g. IL-4↑, IL-10↑, IFN-γ↑)	(Dodd *et al.*, 2013)
Macrolides/tetracyclines	++	IL-1*β*↓, NO↓	(Yrjanheikki *et al*., 1998; Chan and Cronstein, 2010)
Melatonin	++	NO↓, IL-1*β*↓, TNF-*α*↓, NF-*κβ*↓	(Favero *et al*., 2017)
Minocycline	++	Microglia inhibition, IL-1β↓, IL-6↓, TNF-α↓, IFN-γ↓	(Watabe *et al*., 2012; Inta *et al.*, 2017)
Monoclonal antibodies	+/−	Act on specific inflammatory cytokines	(Miller & Buckley, 2016)
N-acetylcysteine	++	IL-1*β*↓, IL-6↓, TNF-*α*↓	(Palacio *et al*., 2011; Liu *et al.*, 2005; Ferreira *et al.*, 2012)
Pioglitazone	++	NF-*κβ*↓	(Iranpour *et al*., 2016)
Piracetam	+/−	IL-1*β*↓, TNF-*α*↓, MPO↓	(Navarro *et al*., 2013)
Pregnenolone	++	IL-6↓, TNF-*α*↓	(Murugan *et al*., 2019)
Statins	++	CRP↓, IL-6↓	(Ridker *et al*., 1999; Asanuma *et al*., 2008; Sierra *et al*., 2011)
Transplantation adjuncts	+/−	Diverse, e.g. IL-1/2/6↓, TNF-*α*↓	(Kotsch *et al*., 2008; Mulders-Manders *et al*., 2017)
Varenicline	++	IL-1*β*↓, TNF-*α*↓, NF-*κβ*↓	(Rosas-Ballina and Tracey, 2009; Kurosawa *et al*., 2017)
Withania somnifera (extract)	++	COX-2↓, NF-*κβ*↓	(Khan *et al*., 2006; Mulabagal *et al*., 2009; Kumar and Patnaik, 2016)

BBB, blood-brain barrier; BDNF, brain-derived neurotrophic factor; CNS, central nervous system; COX, cyclooxygenase; CRP, C-reactive protein; IFN, interferon; IL, interleukin; MPO, myeloperoxidase; MTX, methotrexate; NF-*κβ*, nuclear factor-*κβ*; NO, nitric oxide; PG, prostaglandin; TNF, tumor necrosis factor.

++, excellent BBB crossing; +/−, lower CNS concentrations than in peripheral blood.

a https://www.drugbank.ca/drugs was also accessed to determine the degree of BBB crossing (access date 5 February 2019)

## Methods

### Literature search

The literature was systematically reviewed according to the Preferred Reporting Items for Systematic Reviews and Meta-analyses (Moher *et al*., 2009). Two independent investigators (N.Ç. and G.E.) systematically searched PubMed, Embase, the National Institutes of Health website (http://www.clinicaltrials.gov), and the Cochrane Database of Systematic Reviews from inception to 9 August 2018. No language or year restrictions were applied. The search strategy used for each database can be found in online Supplementary Material S1.

### Inclusion criteria

Consensus on the studies included was reached on the basis of the following criteria:

(1) Randomized, double-blind, placebo-controlled trials regarding augmentation of antipsychotic medication with anti-inflammatory agents.
(2) Patients included had a diagnosis of a schizophrenia spectrum disorder (schizophrenia, schizophreniform disorder, or schizoaffective disorder) according to the diagnostic criteria of the *Diagnostic and Statistical Manual of Mental Disorders* (*DSM-III*, *DSM-III-R*, *DSM-IV*, and *DSM-IV-TR* or *International Classification of Diseases*, 9th or 10th revision). Schizotypal and schizoid personality disorder were not included.
(3) Studies reported information to calculate common effect size (ES) statistics of change scores, i.e. means and standard deviations; exact *p*, *t*, or *z* values; or corresponding authors could supply these data upon request. Studies providing only post-treatment data were not included.

We also included crossover studies to obtain as much information as possible. We excluded antipsychotic, antidepressant, and mood-stabilizing agents because their well-known efficacy on symptom severity would confound the results. Studies that were only published as abstracts were included after contacting the authors for more detailed information. If multiple publications from the same cohort were available, we extracted data from the largest or most recent data set.

### Outcome measures

The primary outcome measure was the mean change in total score on the Positive and Negative Syndrome Scale (PANSS) or the Brief Psychiatric Rating Scale (BPRS). We also investigated effects on PANSS positive, PANSS negative, and cognitive test batteries. Data of the last observation carried forward analysis were used when provided. If only data of completer analyses were given, these data were used instead. The quality of the studies was assessed using the Cochrane risk of bias tool for randomized trials (Higgins *et al*., 2011). Two reviewers (N.Ç. and G.E.) independently extracted data from the papers. Disagreements were resolved by discussion or by a third reviewer (I.E.S.).

### Statistical analyses

We calculated standardized differences from the mean differences (placebo *v.* augmentation) of the change score (end of treatment minus baseline) means and standard deviations (Rosenthal, 1991). When only exact *F* or *p* values for the main effects of the treatment group were presented, these data were used. We calculated standardized mean differences, represented as Hedges' *g* (Shaddish and Haddock, 1994), using a random-effects model. Inconsistency across studies was assessed with the *I*^2^ statistic (Higgins *et al*., 2003), with values ⩾50% indicating high heterogeneity, and values between 30% and 50% indicating moderate heterogeneity. Potential publication bias was assessed using the Egger test of the intercept if 10 or more studies were analysed for the same anti-inflammatory therapy and represented diagrammatically with funnel plots (Egger *et al*., 1997), as recommended by the Cochrane Collaboration (online Supplementary Figs S1–S3) (Higgins and Green, 2008). Subgroup analyses were performed to investigate the effects of anti-inflammatory medication in distinct patient groups, including first-episode psychosis (FEP), early-phase schizophrenia (duration of illness ⩽ 5 years) and chronic schizophrenia (duration of illness > 5 years). Meta-regression of categorical moderators was performed if at least four studies were available. In turn, meta-regression of continuous moderators was performed if at least six studies were available (Fu *et al*., 2011). Following this rule, we assessed the effects of the following moderators: study quality, illness duration, treatment duration, treatment dose, and baseline severity score (as measured with the PANSS total). Results of meta-analysis and meta-regression with a *p* value < 0.05 were considered significant. Results of multiple testing, using the Bonferroni correction (Haynes, 2013), are presented in addition to uncorrected findings for interpretation of the reader. All analyses were performed using Comprehensive Meta-Analysis version 2.0.

## Results

**Fig. 1.**
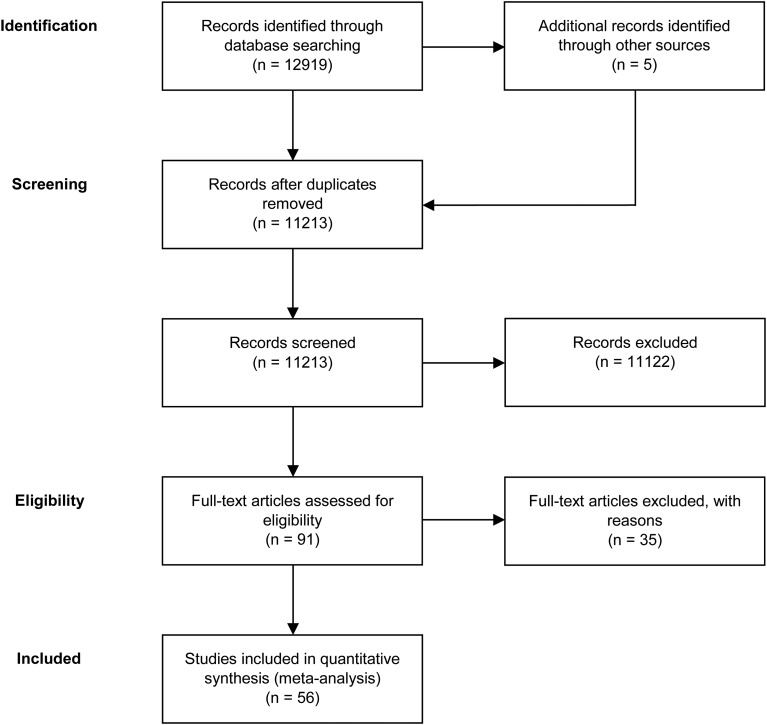
PRISMA flow diagram of the performed literature search.

**Fig. 2.**
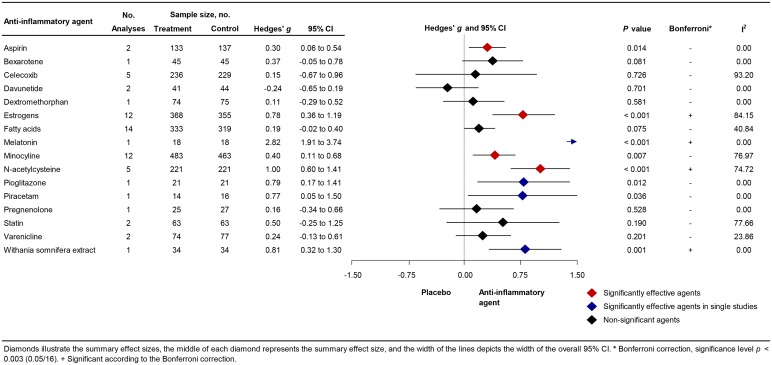
Forest Plot Showing Effect Sizes for Anti-Inflammatory Therapies in Schizophrenia.

A total of 56 studies were retrieved by our search that fulfilled all inclusion criteria (Fig. 1). These studies provided information on the efficacy of the following agents on the improvement of symptom severity in patients with schizophrenia: aspirin, bexarotene, celecoxib, davunetide, dextromethorphan, estrogens, fatty acids including eicosapentaenoic acids (EPA) and docosahexaenoic acids (DHA), melatonin, minocycline, NAC, pioglitazone, piracetam, pregnenolone, statins, varenicline, and withania somnifera extract (WSE). Figure 2 shows the effect sizes of the effects of anti-inflammatory medication on symptom severity. Effect size estimates for individual studies are provided in online Supplementary Figs S4–S19.

Additional study characteristics such as treatment duration and treatment dose are provided in online Supplementary Table S1. A detailed description of the effects of anti-inflammatory agents on positive and negative symptoms can be found in online Supplementary Material S2 and online Supplementary Figs S20–S46. Quality of the studies varied from fair to good quality (online Supplementary Table S2).

### Aspirin

Aspirin is an NSAID that modifies cyclooxygenase-2 (COX-2) activity and irreversibly inhibits cyclooxygenase-1 (COX-1), thereby suppressing the production of prostaglandins and thromboxanes, which are involved in the inflammatory process (Roth and Majerus, 1975; Vane *et al*., 1998). Aspirin also reduces hypothalamic-pituitary-adrenal axis response (Nye *et al*., 1997). The BBB is not readily crossed by aspirin, and aspirin levels in the central nervous system are lower than in peripheral blood (Vasovic *et al*., 2008). Two studies provided 1000 mg aspirin daily to schizophrenia patients in addition to their regular treatment for 3 (Laan *et al*., 2010) or 4 months (Weiser *et al*., 2012). A significant positive influence on total symptom severity was observed [mean weighted effect size (ES): 0.30; 95% confidence interval (CI) 0.06–0.54; *p* = 0.014; heterogeneity (*I*^2^) = 0%].

### Bexarotene

Bexarotene is an antitumor agent that acts via the nuclear retinoid X receptor (RXR) (Lerner *et al*., 2013). Activation of RXR has the potential to increase apolipoprotein E, which inhibits the complement pathway (Tousi, 2015; Yin *et al*., 2019). Bexarotene can easily cross the BBB (Tousi, 2015). One study investigated the effects of bexarotene 75 mg/day for 6 weeks on symptom severity in schizophrenia patients. However, bexarotene did not significantly improve symptom severity (ES: 0.37; CI −0.05 to 0.78; *I*^2^ = 0%).

### Celecoxib

Celecoxib is also an NSAID and has analgesic and inflammatory actions as well. Celecoxib reduces pain and inflammation by blocking COX-2-mediated vascular permeability, thereby reducing extravasation of pro-inflammatory cells, proteins, and enzymes, which enhance the local inflammatory response and lead to edema (Simon, 1999). Celecoxib is a small molecule that can easily cross the BBB (Davies *et al*., 2000). In all five included studies, a dose of 400 mg was provided to schizophrenia patients, and duration of treatment varied from 5 to 11 weeks (Muller *et al*., 2002, 2010; Rappard and Muller, 2004; Rapaport *et al*., 2005; Akhondzadeh *et al*., 2007). We observed heterogeneous results, ranging from strong positive to strong negative effects of celecoxib as augmentation therapy. The effects of celecoxib on the symptom severity was not significant (ES: 0.15; CI −0.67 to 0.96), and heterogeneity was high (*I*^2^ = 93%).

### Davunetide

Davunetide is the smallest active element from the activity-dependent neuroprotective protein, which can readily enter the BBB from the blood (Quintana *et al*., 2006). Davunetide can downregulate key inflammatory cytokines (Quintana *et al*., 2006). We included one study that provided davunetide (5 or 30 mg daily) as augmentation therapy to patients with chronic schizophrenia for 3 months (Javitt *et al*., 2012). Neither dose improved symptom severity (ES: −0.24; CI −0.65 to 0.19; *I*^2^ = 0%).

### Dextromethorphan

Dextromethorphan, an antitussive drug, has neuroprotective and anti-inflammatory effects by inhibiting overactivation of microglia (Zhang *et al*., 2004). One study provided 60 mg dextromethorphan daily in addition to standard treatment to patients with schizophrenia for 11 weeks (Lee *et al*., 2015). Dextromethorphan did not improve symptom severity (ES: 0.11; CI −0.29 to 0.52; *I*^2^ = 0%).

### EPA and DHA fatty acids

Fatty acids, especially EPA and DHA fatty acids, have several mild anti-inflammatory effects, such as decreasing levels of serum IL-1*β*, tumor necrosis factor alpha (TNF-*α*) and interferon-*γ* levels, and neuroprotective effects (Solfrizzi *et al*., 2010; Calder, 2012). Fatty acids also enhance synaptic plasticity and membrane fluidity and affect dopaminergic, serotonergic, and glutamatergic neurotransmission (Glantz and Lewis, 2000; Calder, 2012). Eleven studies were included, of which seven studies added EPA, one study added DHA, and four studies added omega-3 fatty acids (i.e. combination of EPA and DHA) to antipsychotic treatment for patients with schizophrenia (Fenton *et al*., 2001; Peet *et al*., 2001; Peet and Horrobin, 2002; Emsley *et al*., 2002, 2006, 2014; Berger *et al*., 2007; Bentsen *et al*., 2013; Jamilian *et al*., 2014; Boskovic *et al*., 2016; Pawelczyk *et al*., 2016). Daily treatment doses of fatty acids varied (EPA 0.5 g to 4 g, DHA 2 g, omega-3 0.4 g to 2.2 g) as did treatment duration across the studies (8 weeks to 2 years). We observed a trend toward beneficial results for treatment with EPA and/or DHA fatty acids (ES: 0.19; CI −0.02 to 0.40; *p* = 0.075; *I*^2^ = 41%), without indication of publication bias (Egger test *p* = 0.45). One study reported a large negative ES of −0.64 and was regarded as an outlier in an additional analysis (Bentsen *et al*., 2013). Exclusion of this outlier yielded a mean weighted ES of 0.23, which was significant (CI 0.05–0.41; *p* = 0.012; *I*^2^ = 9%). Subgroup analysis showed a trend toward beneficial effects for FEP patients (ES: 0.31; CI −0.02 to 0.64; *p* = 0.064) (online Supplementary Table S3).

### Estrogens

Estrogens, especially 17*β*-estradiol, have immunomodulatory effects by, e.g. regulating innate immune signalling pathways and modulating inflammatory elements such as cytokines (Medina-Estrada *et al*., 2018). Other properties of estrogens include reducing antioxidative stress, controlling energy balance and glucose homeostasis, and influencing dopaminergic neurotransmission (Liu *et al*., 2005). Eleven studies provided estrogen as augmentation therapy for patients with schizophrenia (Kulkarni *et al*., 2001, 2008, 2011, 2016; Akhondzadeh *et al*., 2003; Louza *et al*., 2004; Ghafari *et al*., 2013; Kianimehr *et al*., 2014; Khodaie-Ardakani *et al*., 2015; Usall *et al*., 2016; Weiser *et al*., 2017). Nine studies included only females, and two studies included only males (Kianimehr *et al*., 2014; Khodaie-Ardakani *et al*., 2015). Four studies applied (ethinyl) estradiol (Kulkarni *et al*., 2001, 2008, 2011; Akhondzadeh *et al*., 2003), two studies applied conjugated estrogen (Louza *et al*., 2004; Ghafari *et al*., 2013), and five studies applied raloxifene, a selective estrogen receptor modulator (Kianimehr *et al*., 2014; Khodaie-Ardakani *et al*., 2015; Kulkarni *et al*., 2016; Usall *et al*., 2016; Weiser *et al*., 2017). Estrogen doses ranged from 0.05 mg per day (patch) to 2 mg per day (orally), and raloxifene doses varied from 60 mg to 120 mg per day (orally). One study reported a large ES of 3.7 and was regarded as an outlier (Ghafari *et al*., 2013). Exclusion of this outlier yielded a mean weighted ES of 0.57, which was significant (CI 0.25–0.90; *p* = 0.001; *I*^2^ = 74%). Indication of publication bias was found (Egger test *p* = 0.001). A significant ES was also found when we restricted analyses to female studies only (ES: 0.52; CI 0.18–0.87; *p* = 0.003; *I*^2^ = 72%).

### Melatonin

Melatonin is a multifunctional hormone largely derived from the pineal gland at night under normal light and dark conditions. It is an antioxidant and also a widespread anti-inflammatory molecule, modulating both pro- and anti-inflammatory cytokines, which can easily pass the BBB (Favero *et al*., 2017). One study investigated the effects of adding 3 mg melatonin daily to regular antipsychotic treatment for patients with schizophrenia for 8 weeks (Modabbernia *et al*., 2014). Melatonin showed significant beneficial results on decreasing symptom severity in schizophrenia (ES: 2.82; CI 1.91–3.74; *p* < 0.001; *I*^2^ = 0%).

### Minocycline

Minocycline is a broad-spectrum tetracycline antibiotic that has strong inhibitory effects on microglia cells and can easily cross the BBB (Watabe *et al*., 2012). Ten studies assessed the effect of minocycline augmentation therapy for schizophrenia patients (Levkovitz *et al*., 2010; Chaudhry *et al*., 2012; Ghanizadeh *et al*., 2014; Khodaie-Ardakani *et al*., 2014; Liu *et al*., 2014; Chaves *et al*., 2015; Kelly *et al*., 2015; Deakin *et al*., 2018; Zhang *et al*., 2018; Weiser *et al*., 2019). The daily treatments doses varied from 100 to 300 mg, and the duration of treatment was relatively long, ranging from 2 to 12 months. Minocycline treatment in addition to regular antipsychotic treatment showed significantly beneficial results on symptom severity (ES: 0.40; CI 0.11–0.68; *p* = 0.007; *I*^2^ = 77%), with an indication of publication bias (Egger test *p* < 0.001). One study reported a large negative ES of −0.24 (Deakin *et al*., 2018). Excluding this study from the analysis yielded a mean weighted ES of 0.47 (CI 0.18–0.76; *p* = 0.002; *I*^2^ = 72%). Subgroup analysis showed a trend toward positive effects for patients with early-phase schizophrenia (ES: 0.38; CI −0.02 to 0.78; *p* = 0.060) (online Supplementary Table S3).

### N-acetylcysteine

NAC has evident anti-inflammatory properties and can modulate immune functions during the inflammatory response by inhibiting TNF-*α*, IL-1*β*, and IL-6 (Palacio *et al*., 2011). NAC can also easily pass the BBB (Farr *et al*., 2003). Five studies investigated the effects of NAC augmentation therapy on symptom severity of patients with schizophrenia (Berk *et al*., 2008; Farokhnia *et al*., 2013; Zhang *et al*., 2015; Breier *et al*., 2018; Sepehrmanesh *et al*., 2018). Only one of those studies restricted inclusion to FEP patients only (Zhang *et al*., 2015). Treatment doses varied from 600 mg to 3600 mg, and duration of treatment varied from 8 to 52 weeks. NAC as augmentation therapy had significant beneficial effects on decreasing symptom severity in patients with schizophrenia compared with controls (ES: 1.00; CI 0.60–1.41; *p* < 0.001; *I*^2^ = 75%). Subgroup analysis showed that augmentation therapy with NAC is beneficial in all illness stages, including FEP which yielded the largest ES (ES: 1.42; CI 1.02–1.81; *p* < 0.001), early-phase schizophrenia (ES: 0.98; CI 0.45–1.51; *p* < 0.001), and chronic schizophrenia (ES: 0.44; CI 0.11–0.77; *p* = 0.010) (online Supplementary Table S3).

### Pioglitazone

Pioglitazone is an antidiabetic agent with antioxidant and anti-inflammatory actions (Iranpour *et al*., 2016), and it can cross the BBB (Grommes *et al*., 2013). One study provided 30 mg pioglitazone daily in addition to standard treatment for 8 weeks to patients with schizophrenia (Iranpour *et al*., 2016). Pioglitazone showed significant beneficial results on reducing symptom severity (ES: 0.79; CI 0.17–1.41; *p* = 0.012; *I*^2^ = 0%).

### Piracetam

Piracetam is a nootropic analgesic agent and has anti-inflammatory effects. It can reduce TNF-*α*, IL-1*β*, and myeloperoxidase. There is some evidence that piracetam can cross the BBB (Brust, 1989). One study provided 3200 mg piracetam in addition to regular antipsychotic treatment for 8 weeks to schizophrenia patients (Noorbala *et al*., 1999). A significant positive influence on total symptom severity was observed (ES: 0.77; CI 0.05 to 1.50; *p* = 0.036; *I*^2^ = 0%).

### Pregnenolone

Pregnenolone is a steroid hormone precursor that regulates neuron growth and cerebral BDNF levels (Naert *et al*., 2007; Murugan *et al*., 2019). Pregnenolone is also an anti-inflammatory molecule that can maintain immune homeostasis in various inflammatory conditions (Murugan *et al*., 2019). Pregnenolone can readily cross the BBB (Sripada *et al*., 2013). One study was included that added 50 mg pregnenolone to standard treatment for early-phase schizophrenia patients for 8 weeks (Ritsner *et al*., 2014). For this study, we observed no beneficial effects on the symptom severity (ES: 0.16; CI −0.34 to 0.67; *I*^2^ = 0%).

### Statins

Statins are usually provided as primary or secondary prevention of cardiovascular diseases. Statins also have anti-inflammatory effects by reducing atherogenesis and, concomitantly, inflammation (Pearson *et al*., 2009). Statins can reduce levels of CRP and IL-6, and improve insulin resistance (Ridker *et al*., 1999; Guclu *et al*., 2004; Asanuma *et al*., 2008). The fat-soluble statins can easily cross the BBB (Sierra *et al*., 2011). Two studies provided statins in addition to regular antipsychotic treatment for patients with schizophrenia (Vincenzi *et al*., 2014; Tajik-Esmaeeli *et al*., 2017). Tajik-Esmaeeli *et al*. applied 40 mg simvastatin daily for 8 weeks and Vincenzi *et al*. applied 40 mg pravastatin for 12 weeks. However, beneficial effects on symptom severity were not observed (ES: 0.50; CI −0.25 to 1.25; *I*^2^ = 78%).

### Varenicline

Varenicline is a high-affinity partial agonist at *α*7 nicotinic acetylcholine receptors (nAChRs) and is used to treat nicotine addiction. Varenicline can readily cross the BBB (Kurosawa *et al*., 2017). Activation of the vagus nerve reduces the production of pro-inflammatory cytokines from macrophages, such as TNF-*α*, in the spleen through a mechanism dependent on nAChRs (Rosas-Ballina and Tracey, 2009). It has been shown that varenicline administration reduces brain inflammation and promotes recovery of function following experimental stroke (Chen *et al*., 2017). Two studies provided varenicline in addition to antipsychotic treatment for patients with schizophrenia (Hong *et al*., 2011; Smith *et al*., 2016). Smith *et al*. applied 4 mg varenicline daily for 8 weeks and Hong *et al*. applied 1 mg varenicline for 8 weeks. No beneficial effects were observed on symptom severity (ES: 0.24; CI −0.13 to 0.61; *I*^2^ = 24%).

### Withania somnifera extract

WSE, mostly used as a medicinal herb in Ayurvedic medicine, has anti-inflammatory actions (i.e. inhibition of NF-*κβ* inflammatory signalling pathways and COX-2) (Khan *et al*., 2006; Mulabagal *et al*., 2009). WSE consists of various phytochemicals, of which the effects of 1000 mg withaferin A on symptom severity was investigated in one study for 12 weeks (Chengappa *et al*., 2018). WSE with drug ligand withaferin A can readily cross the BBB (Kumar and Patnaik, 2016). A significant positive influence on total symptom severity was observed (ES: 0.81; CI 0.32–1.30; *p* = 0.001; *I*^2^ = 0%).

### Effects of moderators

Meta-regression analysis showed that illness duration, treatment duration, treatment dose, and baseline severity were insignificant predictors of the ES estimates for the effects of augmentation with EPA and/or DHA fatty acids, estrogen and minocycline (online Supplementary Table S3). Study quality was not a significant moderator for the celecoxib, EPA and/or DHA fatty acids, estrogen, minocycline and NAC studies.

### Cognition

Eighteen studies investigated the effects of anti-inflammatory agents on cognition (online Supplementary Table S4). Heterogeneity of the cognitive tests used across the studies was too great to make a quantitative review of these effects. Notwithstanding, it seemed that minocycline improved attention, executive functions and memory (Levkovitz *et al*., 2010; Liu *et al*., 2014), whereas davunetide (the 5 mg group) improved verbal learning and memory (Javitt *et al*., 2012). NAC (Sepehrmanesh *et al*., 2018) improved attention, memory, and executive functions. However, other studies did not observe any beneficial effects on cognition for minocycline (Chaudhry *et al*., 2012; Kelly *et al*., 2015; Deakin *et al*., 2018; Weiser *et al*., 2019) and NAC (Breier *et al*., 2018). For statins, only one study investigated the effects of pravastatin on cognition and did not observe any significant effects (Vincenzi *et al*., 2014). For varenicline, no cognitive improvement was observed by Smith *et al*. (2016). For the anti-inflammatory components bexarotene, celecoxib, dextromethorphan, melatonin, pioglitazone, piracetam, pregnenolone, and WSE no data on cognitive effects were reported.

## Discussion

In this meta-analysis, we quantitively reviewed the efficacy of various anti-inflammatory medications to reduce symptom severity in patients with schizophrenia. We could include data from 56 studies applying 16 different agents in addition to antipsychotic treatment. The results of aspirin, estrogens, minocycline, and NAC showed significantly better results than placebo in meta-analysis of at least two studies, while pioglitazone, piracetam, and WSE were significant in single studies. Bexarotene, celecoxib, davunetide, dextromethorphan, fatty acids, pregnenolone, statins, and varenicline showed no significant beneficial effects.

### Effects on symptom severity of specific components

Aspirin was found to have beneficial effects on symptom severity in our current study. It is important to note that aspirin has broadly active substances, and it is unclear whether the beneficial effects of aspirin are solely due to its anti-inflammatory properties. Celecoxib, which is a more specific anti-inflammatory agent, showed no beneficial effects. Another meta-analysis found that celecoxib improved symptoms in FEP patients but not in chronic patients (Zheng *et al*., 2017).

Fatty acids as augmentation therapy for patients with schizophrenia showed borderline significant effects on decreasing symptom severity in the current study. However, the included studies showed great heterogeneity in the methods of treatment. Researchers investigated the addition of different fatty acids (i.e. EPA or DHA) or a combination of fatty acids (i.e. EPA and DHA combined). Furthermore, three research groups added antioxidants to the fatty acids treatment regime (Bentsen *et al*., 2013; Emsley *et al*., 2014; Boskovic *et al*., 2016). So, in fact, several different treatment conditions are investigated under the umbrella term ‘fatty acids augmentation’. We also point out that, considering fatty acids augmentation (without anti-oxidants), the results showed a negative association, but this result was greatly influenced by a substantial outlier. Excluding this outlier showed a positive significant association. Furthermore, we observed that FEP patients might benefit the most from treatment with fatty acids compared with patients with a longer illness duration.

In summary, based on the available data, a clear statement about the efficacy of fatty acids, either alone or in combination with anti-oxidants cannot be made yet. Possibly, fatty acids can be beneficial, but the field is still investigating what specific combination of fatty acids is efficacious, and whether or not antioxidants are beneficial. Further research is warranted before a clear recommendation can be made.

Estrogen augmentation therapy for schizophrenia patients showed beneficial effects for a relatively short duration of treatment (starting at 4 weeks). Estrogens act on different ways in the brain and may cause their beneficial effects by mechanisms that are not related to inflammation (e.g. by affecting angiotensin and neurotransmission) (O'Dell *et al*., 1997; Sanchez *et al*., 2012).

Minocycline has strong inhibitory effects on microglia cell activation and may, therefore, be expected to have potential as augmentation therapy for schizophrenia (Inta *et al*., 2017). Microglia activation plays an important role during brain development, but excessive microglia activation is also considered a hallmark of neuroinflammation (Inta *et al*., 2017). Complex variations were found in the complement component 4A (C4A) gene in schizophrenia patients. Human C4 protein is localized to neuronal synapses, axons, dendrites, and cell bodies. These results of high complement activity in the development of schizophrenia could explain the reduced numbers of synapses in the brains of patients with schizophrenia (Sekar *et al*., 2016).

In the current meta-analysis, we found a clear positive result on amelioration of symptom severity and especially in early-phase schizophrenia. However, it should be noted that a large negative study was part of our analysis which provided almost 22% of the total amount of patients (Deakin *et al*., 2018). Deakin and colleagues investigated first-episode patients with an illness duration shorter than 5 years. Minocycline seems to have great beneficial effects on improving negative symptoms in schizophrenia (online Supplementary Fig. S40). We noted that the study population studied by Deakin and colleagues had relatively low baseline levels of PANSS negative symptoms (±17) compared with other studies investigating early-phase schizophrenia patients (>22).

NAC has clear anti-inflammatory and immune-modulating actions. All five studies included in this meta-analysis showed beneficial effects on improving symptom severity. Only one study restricted inclusion to FEP patients and yielded the largest beneficial effects on symptom severity (Zhang *et al*., 2015).

Simvastatin showed beneficial effects on improvement of symptom severity (Tajik-Esmaeeli *et al*., 2017), while pravastatin showed no positive significant effects on symptom severity. The difference can be explained by the fact that simvastatin easily crosses the BBB, while pravastatin does not. We found no significant effects of statins on symptom severity when these two studies were combined. More studies are needed to assess the efficacy of statins, especially of fat-soluble statins on symptom severity in schizophrenia.

### Effects on cognition

The variety in cognitive assessment tests across the 18 studies that investigated the effects of anti-inflammatory medication on cognition was large. We observed that minocycline, NAC, and davunetide could have some cognitive enhancing properties but future research is needed.

### Side effects

Reconsidering the five agents that showed positive results in a meta-analysis of at least two studies, it is worthwhile to consider the side effects of these anti-inflammatory agents. Aspirin use increases the risk of gastrointestinal bleeding and should, therefore, be combined with gastric protection. This serious side effect does not happen infrequently and, therefore, should be considered and monitored. On the other hand, aspirin also possesses cardioprotective properties, which can be beneficial in schizophrenia patients with metabolic syndrome.

Estrogens are not safe for a longer treatment duration than 1–2 months unless combined with progesterone. Estrogens such as raloxifene are sometimes accompanied by hot flashes and gastrointestinal problems. There are potential risks for the occurrence of thromboembolic events and fatal stroke in women with or at increased risk for cardiovascular disease. Therefore, the clinical risk for thromboembolic events should be evaluated and monitored during treatment (Barrett-Connor *et al*., 2006; Adomaityte *et al*., 2008).

Fatty acids are usually well tolerated. There are some reported side effects during administration such as gastrointestinal effects (e.g. constipation or diarrhea) and infection (e.g. upper respiratory infection). The omega-3 fatty acid and anti-oxidant combination might be beneficial (Bentsen *et al*., 2013; Bentsen and Landro, 2018).

NAC is a well-tolerated drug that can also be administered during pregnancy. NAC has other beneficial effects in schizophrenia, such as attenuating addiction (Gipson, 2016) and given that it is a free radical scavenger (Markoutsa and Xu, 2017). The NAC-varenicline combination may be beneficial in schizophrenia (Koola, 2018).

Minocycline is a tetracyclic antibiotic that can be given to a diverse group of patients with schizophrenia. In the included studies, no serious adverse events were observed in the treatment groups.

### Limitations

An important limitation is that many anti-inflammatory augmentation treatment strategies have not been sufficiently investigated. Components with strong anti-inflammatory potency, such as glucocorticosteroids, have not been applied yet to patients with schizophrenia. Also, for most anti-inflammatory medications a limited number of studies was available. Most studies did not stratify schizophrenia patients in subgroups of illness duration. Furthermore, there was an insufficient description of signs of inflammation before the start of anti-inflammatory therapy. For designing future research it would be interesting to investigate whether signs of (low-grade) inflammation before the start of the trials would influence the outcome and degree of inflammation. There is increasing evidence from the biomarker research field that cytokine alterations are already present from disease-onset (Schwarz *et al*., 2014; Upthegrove *et al*., 2014; van Beveren *et al*., 2014). It would be interesting for further trials to stratify patients according to the presence of immune alterations and to investigate which inflammatory subtypes would benefit the most from anti-inflammatory therapy. This opens up the way for personalized medicine based on inflammatory markers.

## Conclusion

The anti-inflammatory medications aspirin, estrogens, minocycline, and NAC improved symptom severity in patients with schizophrenia. We observed greater beneficial results in early-psychosis studies. Evidence for cognitive improvement is scarce. Taken together, there is evidence for the efficacy of some anti-inflammatory agents on symptom severity in schizophrenia which could confirm the immune hypothesis in schizophrenia, but further studies are still needed.

## Supplementary material

For supplementary material accompanying this paper visit https://doi.org/10.1017/S0033291719001995. click here to view supplementary material
