# Supplementary material for: An update on the efficacy of anti-inflammatory agents for patients with schizophrenia: a meta-analysis
Source: Psychol Med. 2019 Aug 23;49(14):2307–19. doi: 10.1017/S0033291719001995 (PMC6763537; doi:10.1017/S0033291719001995)
Supplement: Supplementary file 1 [file S0033291719001995sup001.docx]

**Supplementary Material**

**Index**

Supplementary Material 1. Search strategy *Pages 2-3*

Supplementary Material 2. Effects of anti-inflammatory agents on *Page 4*

positive and negative symptoms

Supplementary Table 1. Baseline characteristics *pages 5-9*

Supplementary Table 2. Results of the Cochrane risk of bias tool for *pages 10-11*
randomized trials

Supplementary Table 3. Subgroup analysis and moderators *Page 12*

Supplementary Table 4. Cognitive domains *Pages 13-14*

Supplementary Table 5. Meta-analysis of RCTs with aspirin, estrogens, *Page 15*

minocycline, and *N*-acetylcysteine in schizophrenia: efficacy
signal

Supplementary Figures 1-3. Funnel plots *Pages 16-17*

Supplementary Figures 4-19. Total symptom severity *Pages 18-28*

Supplementary Figures 20-33. Positive symptoms *Pages 29-35*

Supplementary Figures 34-46. Negative symptoms *Pages 36-42*

**Supplementary Material 1.** Search strategy

**PubMed**

("Schizophrenia Spectrum and Other Psychotic Disorders"[Mesh] OR "Psychotic Disorders"[Mesh] OR "Schizophrenia"[Mesh] OR schizophren*[tiab] OR psychosis[tiab] OR psychot*[tiab] OR schizoaffective[tiab]) AND (“Anti-Inflammatory Agents"[Mesh] OR antiinflammatory[tiab] OR Anti-Inflammatory[tiab] OR "Anti-Inflammatory Agents, Non-Steroidal"[Mesh] OR NSAID*[tiab] OR "Aspirin"[Mesh] OR aspirin*[tiab] OR "Celecoxib"[Mesh] OR celecoxib[tiab] OR "Ibuprofen"[Mesh] OR ibuprofen[tiab] OR "Diclofenac"[Mesh] OR diclofenac[tiab] OR "Naproxen"[Mesh] OR naproxen[tiab] OR davunetide[tiab] OR "Fatty Acids"[Mesh] OR fatty acid*[tiab] OR "Estrogens"[Mesh] OR estrogen*[tiab] OR 17β-estradiol[tiab] OR raloxifene[tiab] OR "Tamoxifen"[Mesh] OR tamoxifen[tiab] OR acetylcysteine*[tiab] OR "Acetylcysteine"[Mesh] OR N-acetylcysteine*[tiab] OR NAC[tiab] OR corticosteroid*[tiab] OR "Prednisone"[Mesh] OR prednisone[tiab] OR "Prednisolone"[Mesh] OR prednisolone*[tiab] OR "Hydrocortisone"[Mesh] OR hydrocortisone*[tiab] OR "methylprednisolone"[Mesh] OR methylprednisolone*[tiab] OR "dexamethasone"[Mesh] OR dexamethasone*[tiab] OR "Dexmedetomidine"[Mesh] OR dexmedetomidine[tiab] OR "Cortisone"[Mesh] OR cortisone*[tiab] OR "Triamcinolone"[Mesh] OR triamcinolone*[tiab] OR "Betamethasone"[Mesh] OR betamethasone[tiab] OR "Tacrolimus"[Mesh] OR tacrolimus[tiab] OR "Cyclosporine"[Mesh] OR "Cyclosporins"[Mesh] OR cyclosporine[tiab] OR cyclosporins[tiab] OR "Everolimus"[Mesh] OR everolimus[tiab] OR sirolimus[tiab] OR mycophenolate mofetil[tiab] OR "Cytostatic Agents"[Mesh] OR cytostatic*[tiab] OR bexarotene[tiab] OR "Bone Marrow Transplantation"[Mesh] OR bone marrow irradiation*[tiab] OR bone marrow transplantation*[tiab] OR "Methotrexate"[Mesh] OR methotrexate[tiab] OR "Cyclophosphamide"[Mesh] OR cyclophosphamide[tiab] OR "Melatonin"[Mesh] OR melatonin*[tiab] OR "Ketamine"[Mesh] OR ketamine[tiab] OR parecoxib[tiab] OR "Erythromycin"[Mesh] OR "Piracetam"[Mesh] OR piracetam[tiab] OR statins[tiab] OR "Pregabalin"[Mesh] OR pregabalin[tiab] OR "Lidocaine"[Mesh] OR lidocaine*[tiab] OR "Propofol"[Mesh] OR propofol[tiab] OR "Thiopental"[Mesh] OR thiopental[tiab] OR "Xenon"[Mesh] OR xenon[tiab] OR "Magnesium"[Mesh] OR magnesium[tiab] OR "Erythropoietin"[Mesh] OR erythropoietin*[tiab] OR erythromycin[tiab] OR "Minocycline"[Mesh] OR minocycline[tiab] OR "Anti-Bacterial Agents"[Mesh] OR antibiotic*[tiab]))

Publication year restriction: 2012-2018.

**PubMed**

("Schizophrenia Spectrum and Other Psychotic Disorders"[Mesh] OR "Psychotic Disorders"[Mesh] OR "Schizophrenia"[Mesh] OR schizophren*[tiab] OR psychosis[tiab] OR psychot*[tiab] OR schizoaffective[tiab]) AND (OR 17β-estradiol[tiab] OR "Ketamine"[Mesh] OR ketamine[tiab] OR parecoxib[tiab] OR "Erythromycin"[Mesh] OR "Piracetam"[Mesh] OR piracetam[tiab] OR statins[tiab] OR "Pregabalin"[Mesh] OR pregabalin[tiab] OR "Lidocaine"[Mesh] OR lidocaine*[tiab] OR "Propofol"[Mesh] OR propofol[tiab] OR "Thiopental"[Mesh] OR thiopental[tiab] OR "Xenon"[Mesh] OR xenon[tiab] OR "Magnesium"[Mesh] OR magnesium[tiab] OR "Erythropoietin"[Mesh] OR erythropoietin*[tiab] OR erythromycin[tiab] OR "Anti-Bacterial Agents"[Mesh] OR antibiotic*[tiab varenicline[tiab] OR "Varenicline"[Mesh]))

Publication year restriction: none.

**Embase**

'schizophrenia spectrum disorder' OR schizophrenia OR 'schizophreniform disorder' OR 'paranoid psychosis' OR 'paranoid schizophrenia' OR psychosis OR 'schizoaffective psychosis'

schizophren* or psychotic or psychosis.ab,kw,ti

Anti-inflammatory OR Antiinflammatory OR NSAID* OR aspirin* OR celecoxib OR ibuprofen OR diclofenac OR naproxen OR davunetide OR fatty acid* OR estrogen* OR 17β-estradiol OR raloxifene OR tamoxifen OR acetylcysteine* OR N-acetylcysteine* OR NAC OR corticosteroid* OR prednisone OR prednisolone* OR hydrocortisone* OR methylprednisolone* OR dexamethasone* OR dexmedetomidine OR cortisone* OR triamcinolone* OR betamethasone OR tacrolimus OR cyclosporine OR cyclosporins OR everolimus OR sirolimus OR mycophenolate mofetil OR cytostatic* OR bexarotene OR bone marrow irradiation* OR bone marrow transplantation* OR methotrexate OR cyclophosphamide OR melatonin* OR ketamine OR parecoxib OR piracetam OR statins OR pregabalin OR lidocaine* OR propofol OR thiopental OR xenon OR magnesium OR erythropoietin* OR erythromycin OR minocycline OR antibiotic.ab,kw,sh,ti

Publication year restriction: 2012-2018.

**Embase**

'schizophrenia spectrum disorder' OR schizophrenia OR 'schizophreniform disorder' OR 'paranoid psychosis' OR 'paranoid schizophrenia' OR psychosis OR 'schizoaffective psychosis'

schizophren* or psychotic or psychosis.ab,kw,ti

17β-estradiol cyclosporine OR ketamine OR parecoxib OR piracetam OR statins OR pregabalin OR lidocaine* OR propofol OR thiopental OR xenon OR magnesium OR erythropoietin* OR erythromycin OR antibiotic OR varenicline.ab,kw,sh,ti

Publication year restriction: none.

**The National Institutes of Health website: http://www.clinicaltrials.gov**

'schizophrenia spectrum disorder' OR schizophrenia OR 'schizophreniform disorder' OR 'paranoid psychosis' OR 'paranoid schizophrenia' OR psychosis OR 'schizoaffective psychosis'

Anti-inflammatory OR Antiinflammatory OR NSAID* OR aspirin* OR celecoxib OR ibuprofen OR diclofenac OR naproxen OR davunetide OR fatty acid* OR estrogen* OR 17β-estradiol OR raloxifene OR tamoxifen OR acetylcysteine* OR N-acetylcysteine* OR NAC OR corticosteroid* OR prednisone OR prednisolone* OR hydrocortisone* OR methylprednisolone* OR dexamethasone* OR dexmedetomidine OR cortisone* OR triamcinolone* OR betamethasone OR tacrolimus OR cyclosporine OR cyclosporins OR everolimus OR sirolimus OR mycophenolate mofetil OR cytostatic* OR bexarotene OR bone marrow irradiation* OR bone marrow transplantation* OR methotrexate OR cyclophosphamide OR melatonin* OR ketamine OR parecoxib OR piracetam OR statins OR pregabalin OR lidocaine* OR propofol OR thiopental OR xenon OR magnesium OR erythropoietin* OR erythromycin OR minocycline OR antibiotic*

Publication year restriction: none.

**Cochrane Reviews**

"Schizophrenia" OR "Psychotic " OR schizophren* OR psychosis OR psychot* OR schizoaffective

AND

Anti-inflammatory OR Antiinflammatory OR NSAID* OR aspirin* OR celecoxib OR ibuprofen OR diclofenac OR naproxen OR davunetide OR fatty acid* OR estrogen* OR 17β-estradiol OR raloxifene OR tamoxifen OR acetylcysteine* OR N-acetylcysteine* OR NAC OR corticosteroid* OR prednisone OR prednisolone* OR hydrocortisone* OR methylprednisolone* OR dexamethasone* OR dexmedetomidine OR cortisone* OR triamcinolone* OR betamethasone OR tacrolimus OR cyclosporine OR cyclosporins OR everolimus OR sirolimus OR mycophenolate mofetil OR cytostatic* OR bexarotene OR bone marrow irradiation* OR bone marrow transplantation* OR methotrexate OR cyclophosphamide OR melatonin* OR ketamine OR parecoxib OR piracetam OR statins OR pregabalin OR lidocaine* OR propofol OR thiopental OR xenon OR magnesium OR erythropoietin* OR erythromycin OR minocycline OR antibiotic*

Publication year restriction: 2012-2018.

**Cochrane Reviews**

"Schizophrenia" OR "Psychotic " OR schizophren* OR psychosis OR psychot* OR schizoaffective

AND

17β-estradiol OR ketamine OR parecoxib OR piracetam OR statins OR pregabalin OR lidocaine* OR propofol OR thiopental OR xenon OR magnesium OR erythropoietin* OR erythromycin OR antibiotic*

Publication year restriction: none.

Note: this search strategy was an update from a previously published study (Sommer, I. E., van Westrhenen, R., Begemann, M. J., de Witte, L. D., Leucht, S., Kahn, R. S. (2014). Efficacy of anti-inflammatory agents to improve symptoms in patients with schizophrenia: an update. Schizophrenia bulletin 40, 181-91).

**Supplementary Material** **2.** Effects of anti-inflammatory agents on positive and negative symptoms

The results of aspirin, estrogens, minocycline, and *N-*acetylcysteine (NAC) were significant in meta-analysis of at least two studies. An overview is presented in Supplementary Table 5.

*Effects on positive symptoms*

Positive symptoms were improved by the anti-inflammatory agents estrogens (ES: 0.48; 95% CI, 0.15 to 0.81; *p*=0.004; I2=76%) and celecoxib (ES: 0.54; 95%CI, 0.22 to 0.85; *p*=0.001; I2=0%). Bexarotene, melatonin, and pioglitazone seem to have promising effects on improving positive symptoms, but were investigated in single studies only (Supplementary Figures 20–33).

*Effects on negative symptoms*

Negative symptoms are relatively resistant to antipsychotics and are associated with worse social functioning (Howes *et al.* 2017). It is therefore important, to consider the possibility of additional treatment strategies which could treat negative symptoms. Augmentation therapy with minocycline (ES: 0.50; 95% CI, 0.17 to 0.84; *p*=0.003; I2=82%), NAC (ES: 0.75; 95% CI, 0.19 to 1.32; *p*=0.009; I2=88%), and estrogen (ES: 0.45; 95% CI, 0.13 to 0.77; *p*=0.006; I2=73%) showed positive results on improving negative symptoms. The following agents were investigated in only single studies and seem to have beneficial effects on negative symptoms: melatonin, pioglitazone, piracetam, pregnenolone, and withania somnifera extract (WSE).

In perspective, we like to point out that additional strategies for improving negative symptoms warrant additional research (Supplementary Figures 34-46).

| **Supplementary Table 1.** Baseline characteristics of the included studies. | | | | | | | | | | | | | | | |
| --- | --- | --- | --- | --- | --- | --- | --- | --- | --- | --- | --- | --- | --- | --- | --- |
|  |  |  |  |  |  |  |  |  |  |  |  |  |  |  |  |
|  |  | **Sample Size, No.** |  | **Males, No. / Females, No.** |  | **Age, Mean (SD)** |  | **Duration of Illness (years)** |  |  |  |  |  | **Baseline Severity, PANSS Total, Mean (SD)** | |
| **Study** | **Anti-Inflammatory Agent** | **Treatment / Placebo** |  | **Treatment / Placebo** |  | **Treatment / Placebo** |  | **Treatment / Placebo** |  | **Daily Treatment Dose** | **Duration of Treatment** | **Illness Stage** |  | **Treatment** | **Placebo** |
| **Laan et al., 201** | Aspirin | 33 / 37 |  | 25/8 / 33/4 |  | 31.6 (8.9) / 30.6 (9.2) |  | 4.1 (3.0) / 3.4 (2.5) |  | 1000 mg | 3 months | Early-phase |  | 71.1 (10.6) | 73.1 (10.3) |
| **Weiser et al., 2012** | Aspirin | 100 / 100 |  | n/a |  | 43.2 (10.5) / 41.4 (10.4) |  | n/a |  | n/a | 12 weeks | n/a |  | n/a | n/a |
| **Lerner et al., 2013** | Bexarotene | 45 / 45 |  | 41/4 / 40/5 |  | 41.2 (12.4) / 41.7 (10.0) |  | n/a |  | 75 mg | 6 weeks | Chronic |  | 73.2 (18.9) | 74.9 (24.0) |
| **Akhondzadeh et al., 2007** | Celecoxib | 30 / 30 |  | 18/12 / 17/13 |  | 33.10 (7.29) / 34.30 (7.21) |  | 7.79 (5.87) / 7.98 (5.87) |  | 400 mg | 6 weeks | Early-phase |  | n/a | n/a |
| **Müller et al., 2002** | Celecoxib | 25 / 25 |  | 14/11 / 11/14 |  | 35.9 (12.8) / 35.5 (13.6) |  | n/a |  | 400 mg | 5 weeks | Early-phase |  | n/a | n/a |
| **Müller et al., 2010** | Celecoxib | 25 / 25 |  | 14/11 / 16/9 |  | 26.2 (7.7) / 30.9 (8.1) |  | 16.0 (5.0) / 14.9 (4.6) [months] |  | 400 mg | 6 weeks | FEP |  | 94.5 (16.2) | 95.9 (19.1) |
| **Rapaport et al., 2005** | Celecoxib | 18 / 17 |  | 16/2 / 13/4 |  | 44.1 (9.2) / 47.3 (11.4) |  | n/a |  | 400 mg | 8 weeks | Early-phase |  | 84.1 (11.4) | 84.2 (12.9) |
| **Rappard & Müller 2004** | Celecoxib | 138 / 132 |  | n/a |  | n/a |  | n/a |  | 400 mg | 11 weeks | Early-phase |  | n/a | n/a |
| **Javitt et al. 2012 (30 mg)*** | Davunetide | 21 / 22 |  | n/a |  | n/a |  | n/a |  | 30 mg | 12 weeks | n/a |  | BPRS: 29.1 (6.1) | BPRS: 29.3 (7.6) |
| **Javitt et al. 2012 (5 mg)*** | Davunetide | 20 / 22 |  | n/a |  | 45.2 (8.2) / 41.4 (10.4) |  | n/a |  | 5 mg | 12 weeks | n/a |  | BPRS: 31.2 (5.5) | BPRS: 29.3 (7.6) |
| **Lee et al., 2015** | Dextromethorphan | 74 / 75 |  | 44/30 / 46/29 |  | 30.6 (8.2) / 30.0 (7.2) |  | n/a |  | 60 mg | 11 weeks | n/a |  | 86.3 (12.6) | 86.9 (14.9) |
| **Akhondzadeh et al., 2003** | Estrogen (ethinyl estradiol) | 16 / 16 |  | 0/16 / 0/16 |  | 32.128 (6.31) / 33.37 (6.72) |  | 96.87 (90.52) / 87.37 (49.13) [months] |  | 0.5 mg | 8 weeks | Chronic |  | n/a | n/a |
| **Ghafari et al., 2013** | Estrogen (conjugated estrogen) | 15 / 15 |  | 0/15 / 0/15 |  | 34.2 (9.1) / 34.8 (8.3) |  | n/a |  | 0.625 mg | 4 weeks | Chronic |  | n/a | n/a |
| **Khodaie-Ardakani et al., 2015** | Estrogen (raloxifene) | 21 / 21 |  | 21/0 / 21/0 |  | 31.4 (5.9) / 32.4 (7.8) |  | 89.3 (70.9) / 96.2 (45.9) [months] |  | 120 mg | 8 weeks | Chronic |  | 101.6 (13.5) | 100.7 (15.5) |
| **Kianimehr et al., 2014** | Estrogen (raloxifene) | 25 / 25 |  | 0/25 / 0/25 |  | 61.96 (4.49) / 60.44 (5.28) |  | 17.24 (12.03) / 13.64 (12.41) |  | 120 mg | 8 weeks | Chronic |  | 105.52 (16.96) | 105.00 (11.68) |
| **Kulkarni et al., 2001 (0.05 mg)*** | Estrogen (transdermal estradiol) | 12 / 12 |  | 0/12 / 0/12 |  | 34.00 (9.2) / 34.91 (7.6) |  | 5.5 (5.3) / 10.33 (8.7) |  | 0.05 mg every 4 days | 4 weeks | Chronic |  | 68.17 (10.8) | 67.58 (14.5) |
| **Kulkarni et al., 2001 (10 mg)*** | Estrogen (transdermal estradiol) | 12 / 12 |  | 0/12 / 0/12 |  | 32.83 (8.6) / 34.91 (7.6) |  | 7.38 (7.1) / 10.33 (8.7) |  | 0.10 mg every 4 days | 4 weeks | Chronic |  | 75.83 (18.8) | 67.58 (14.5) |
| **Kulkarni et al., 2008** | Estrogen (transdermal estradiol) | 56 / 46 |  | 0/56 / 0/46 |  | 33.5 (8.8) / 33.8 (7.7) |  | n/a |  | 0.10 mg every 3.5 days | 28 days | Chronic |  | 77.48 (20.0) | 73.40 (12.5) |
| **Kulkarni et al., 2011 (only men included)** | Estrogen (estradiol valerate) | 26 / 27 |  | 26/0 / 27/0 |  | 32.9 (10.2) / 31.2 (12.4) |  | 7.6 (7.7) / 6.1 (7.1) |  | 2 mg | 14 days | Chronic |  | 72.6 (10.7) | 73.5 (9.2) |
| **Kulkarni et al., 2016** | Estrogen (raloxifene) | 26 / 30 |  | 0/26 / 0/30 |  | 52.92 (8.07) / 53.07 (7.43) |  | 24 (11) |  | 120mg | 12 weeks | Chronic |  | 79.96 (15.91) | 77.03 (14.85) |
| **Louzã et al., 2004** | Estrogen (conjugated estrogen) | 21 / 19 |  | 0/21 / 0/19 |  | 34.1 (7.9) / 30.4 (8.3) |  | n/a |  | 0.625 mg | 4 weeks | Chronic |  | n/a | n/a |
| **Usall et al., 2016** | Estrogen (raloxifene) | 38 / 32 |  | 0/38 / 0/32 |  | 62.03 (9.39) / 61.34 (10.41) |  | n/a |  | 60 mg | 24 weeks | Chronic |  | 80.47 (14.30) | 74.66 (13.26) |
| **Weiser et al., 2017** | Estrogen (raloxifene) | 100 / 100 |  | 0/100 / 0/100 |  | 56.6 (4.6) / 55.8 (4.7) |  | n/a |  | 120 mg | 16 weeks | Chronic |  | 101.7 (18.5) | 101.2 (18.1) |
| **Bentsen et al. 2013 (EPA)*** | Fatty acids (EPA) | 33 / 24 |  | 20/13 / 17/7 |  | 25.7 (5.4) / 28.3 (5.8) |  | Median: 2 (1-5.5) / 7 (2-10) |  | 2000 mg EPA | 16 weeks | n/a |  | 78 (16) | 82.5 (19) |
| **Bentsen et al. 2013 (EPA+vitamins E&C)*** | Fatty acids (EPA) | 19 / 24 |  | 12/7 / 17/7 |  | 27.6 (7.1) / 28.3 (5.8) |  | Median: 3.5 (1-6.5) / 7 (2-10) |  | 2000 mg EPA + 364 mg RRR-α-tocopherol + 1000 mg slow release ascorbic acid | 16 weeks | n/a |  | 94.5 (30) | 82.5 (19) |
| **Berger et al. 2007 (EPA)** | Fatty acids (EPA) | 35 / 34 |  | 25/10 / 28/7 |  | 20.5 (3.8) / 20.6 (3.7) |  | 7.2 (10) / 10.0 (13.1) [months] |  | 2 g | 12 weeks | FEP |  | BPRS: 61.6 (9.6) | BPRS: 62.8 (13.8) |
| **Boskovic et al. 2016 (EPA+vitamin E)*** | Fatty acids (omega-3: EPA, DHA, ALA, OE) | 9 / 11 |  | 4/5 / 4/7 |  | 53.6 (8.7) / 45.6 (8.7) |  | 201 (94) / 190 (116) [months] |  | Omega-3 (EPA 132 mg, DHA 88 mg, ALA 94 mg, and OE 52 mg) | 4 months | Chronic |  | 58 .9 (21.5) | 57.8 (14.2) |
| **Boskovic et al. 2016 (EPA+DHA+ALA+OE)*** | Fatty acids (omega-3: EPA, DHA, ALA, OE) | 9 / 11 |  | 6/3 / 4/7 |  | 54.8 (9.6) / 45.6 (8.7) |  | 184 (18) / 190 (116) [months] |  | Omega-3 + vitamin E 600 IU | 4 months | Chronic |  | n/a | n/a |
| **Emsley et al. 2002 (3g EPA)** | Fatty acids (EPA) | 20 / 20 |  | n/a |  | 46.2 (10.6) / 43.6 (13.9) |  | 23.1 / 22.2 |  | 3 g | 12 weeks | Chronic |  | n/a | n/a |
| **Emsley et al. 2006 (2g EPA)** | Fatty acids (EPA) | 39 / 38 |  | 27/12 / 24/14 |  | 42.4 (10.3) / 43.4 (10.9) |  | 16.0 (10.5) / 16.8 (10.4) |  | 2 g | 12 weeks | Chronic |  | 59.2 (13.0) | 57.5 (11.8) |
| **Emsley et al. 2014 (1g DHA+2g EPA+ α-LA)** | Fatty acids (omega-3) | 21 / 12 |  | 16/5 / 8/4 |  | 30.6 (7.4) / 28.1 (8.9) |  | n/a |  | Omega-3 (EPA 2 g + DHA 1 g + 300 mg α-LA) | 2 years or until relapse | Early-phase |  | 36.1 (4.2) | 38.2 (4.0) |
| **Fenton et al. 2001 (EPA)** | Fatty acids (EPA) | 43 / 44 |  | 53/34 |  | 40 (10) |  | n/a |  | 3 g | 16 weeks | Chronic |  | 74 (16) | 76 (18) |
| **Jamilian et al. 2017 (omega-3)** | Fatty acids (omega-3) | 30 / 30 |  | 16/14 / 15/15 |  | 32.01 (7.13) / 31.01 (8.81) |  | n/a |  | 1 g | 8 weeks | Chronic |  | 96.13 (9.61) | 98.26 (4.51) |
| **Pawelcyk et al. 2016 (EPA+DHA)** | Fatty acids (omega-3: EPA and DHA) | 36 / 35 |  | 19/17 |  | 23.2 (4.8) / 23.3 (4.8) |  | 3.1 (4.2) / 2.7 (3.5) [months] |  | Omega-3 2.2g  (EPA 1.32 g and DHA 0.88 g) | 26 weeks | FEP |  | 98.4 (13.22) | 96.8 (12.01) |
| **Peet and Horobin 2002 (EPA)** | Fatty acids (DHA) | 9 / 7 |  | n/a |  | n/a |  | n/a |  | 4 g | 12 weeks | n/a |  | 73.4 (17.9) | 76.2 (20.6) |
| **Peet et al. 2001 (DHA)*** | Fatty acids (EPA) | 16 / 14 |  | 12/4 / 8/6 |  | 42.0 (10.6) / 43.8 (10.8) |  | n/a |  | 2 g | 12 weeks | n/a |  | 73.4 (17.9) | 76.2 (20.6) |
| **Peet et al. 2001 (EPA)*** | Fatty acids (EPA) | 15 / 14 |  | 10/5 / 8/6 |  | 44.2 (11.3) / 43.8 (10.8) |  | n/a |  | 2 g | 12 weeks | n/a |  | 69.9 (12.9) | 76.2 (20.6) |
| **Modabbernia et al., 2014** | Melatonin | 18 / 18 |  | 13/5 / 12/6 |  | 32.7 (7.3) / 32.8 (8.2) |  | n/a |  | 3 mg | 8 weeks | FEP |  | 113.5 (12.7) | 103.5 (18.0) |
| **Chaudhry et al., 2012 (Brazil)** | Minocycline | 15 / 15 |  | 41/30 / 45/28 |  | 25.87 (7.07) / 26.59 (8.26) |  | < 5 |  | start dose: 50 mg; end dose: 200 mg | 8 weeks | Early-phase |  | 63.00 (17.19) | 57.87 (13.25) |
| **Chaudhry et al., 2012 (Pakistan)** | Minocycline | 56 / 58 |  | 41/30 / 45/28 |  | 25.87 (7.07) / 26.59 (8.26) |  | < 5 |  | start dose: 50 mg; end dose: 200 mg | 8 weeks | Early-phase |  | 82.24 (21.55) | 83.84 (20.64) |
| **Chaves et al., 2015** | Minocycline | 16 / 14 |  | 13/3 / 11/3 |  | 24 (5.02) / 25 (6.37) |  | 31.8 (21.98) / 29.1 (17.9) [months] |  | 200 mg | 12 months | Early-phase |  | n/a | n/a |
| **Deakin et al., 2018** | Minocycline | 103 / 103 |  | 77/27 / 73/30 |  | 25.5 (5.2) / 25.7 (5.1) |  | < 5 |  | start dose: 200 mg; end dose: 300 mg | 12 months | Early-phase |  | 67.1 (13.2) | 69.3 (15.4) |
| **Ghanizadeh et al., 2014** | Minocycline | 21 / 22 |  | 15/6 / 19/3 |  | 31.0 (7.6) / 30.2 (8.9) |  | 3.8 (1.7) / 3.2 (1.6) |  | 200 mg | 8 weeks | Early-phase |  | 43.9 (14.9) | 40.3 (10.8) |
| **Kelly et al., 2015** | Minocycline | 27 / 23 |  | 20/8 / 18/5 |  | 42.9 (14.2) / 42.3 (11.0) |  | 24.4 |  | 200 mg | 10 weeks | Chronic |  | BPRS: 44.9 (8.7) | BPRS: 44.0 (7.9) |
| **Khodaie-Ardakani et al., 2014** | Minocycline | 20 / 20 |  | 14/6 / 15/5 |  | 41.05 (7.47) / 38.95 (7.78) |  | 20.90 (8.02) / 18.75 (7.55) |  | start dose: 100 mg; end dose: 200 mg | 8 weeks | Chronic |  | 71.35 (4.54) | 71.90 (7.14) |
| **Levkovitz et al., 2010** | Minocycline | 36 / 18 |  | 3/10 / 3/15 |  | 24.8 (4.01) / 25.5 (4.06) |  | 3.78 / 3.73 |  | 200 mg | 6 months | Early-phase |  | 80.37 (12.77) | 82.86 (13.90) |
| **Liu et al., 2014** | Minocycline | 39 / 40 |  | 25/14 / 24/16 |  | 27.05 (5.68) / 27.70 (7.27) |  | 21.00 (13.84) / 27.45 (14.25) [months] |  | 200 mg | 16 weeks | Early-phase |  | 81.28 (12.88) | 83.35 (10.65) |
| **Weiser et al., 2019** | Minocycline | 100 / 100 |  | n/a |  | 43.4 (10.5) / 43.5 (9.7) |  | 17.2 / 17.2 |  | 200 mg | 16 weeks | Chronic |  | 94.6 (14.3) | 96.5 (16.0) |
| **Zhang et al., 2018 (100 mg minocycline)*** | Minocycline | 25 / 25 |  | 13/12 / 12/13 |  | 33.04 (7.78) / 33.68 (6.18) |  | 6.28 (1.82) / 6.27 (1.71) |  | 100 mg | 3 months | n/a |  | 79.04 (5.04) | 78.08 (4.71) |
| **Zhang et al., 2018 (200 mg minocycline)*** | Minocycline | 25 / 25 |  | 12/13 / 12/13 |  | 33.24 (6.48) / 33.68 (6.18) |  | 5.98 (1.78) / 6.27 (1.71) |  | 200 mg | 3 months | n/a |  | 78.52 (4.58) | 78.08 (4.71) |
| **Berk et al., 2008** | *N*-acetylcysteine | 69 / 71 |  | 48/21 / 50/21 |  | 37.2 (10.1) / 36.1 (11.7) |  | 12.4 (8.2) / 12.1 (9.6) |  | 2000 mg | 24 weeks | Chronic |  | 64.0 (15.4) | 64.4 (16.3) |
| **Breier et al., 2018** | *N*-acetylcysteine | 30 / 30 |  | 23/7 / 24/6 |  | 22.2 (4.2) / 25.0 (5.2) |  | 1.3 (1.2) / 1.4 (1.1) |  | 3600 mg | 52 weeks | Early-phase |  | 56.7 (15.0) | 56.4 (12.0) |
| **Farokhnia et al., 2013** | *N*-acetylcysteine | 21 / 21 |  | 9/12 / 11/10 |  | 32.23 (6.12) / 33.38 (6.97) |  | 83.23 (41.02) / 88.95 (44.66) [months] |  | 2000 mg | 8 weeks | Blank |  | 113.42 (9.05) | 114.61 (10.09) |
| **Sepehrmanesh et al., 2018** | *N*-acetylcysteine | 40 / 39 |  | 22/18 / 19/20 |  | 38.7 (1.9) / 39.4 (2.2) |  | 13.8 (9.9) / 17 (11.6) |  | 1200 mg | 12 weeks | Blank |  | 104.0 (27.0) | 87.7 (17.4) |
| **Zhang et al., 2015** | *N*-acetylcysteine | 61 / 60 |  | 32/29 |  | 34.6 (8.4) |  | 6.6 (5.1) [months] |  | 600 mg | 8 weeks | FEP |  | 113.87 (3.57) | 113.67 (4.36) |
| **Iranpour et al., 2016** | Pioglitazone | 21 / 21 |  | 14/7 / 15/6 |  | 38 (8.99) / 37 (7.69) |  | 16.25 (8.94) / 13.60 (8.21) |  | 30 mg | 8 weeks | n/a |  | 70.45 (5.54) | 68.50 (5.46) |
| **Noorbala et al., 1999** | Piracetam | 14 / 16 |  | 18/16 |  | n/a |  | n/a |  | 3200 mg | 8 weeks | n/a |  | n/a | n/a |
| **Ritsner et al., 2014** | Pregnenolone | 25 / 27 |  | 22/3 / 23/4 |  | 26.9 (5.2) / 27.8 (6.0) |  | 2.5 (1.4) / 2.8 (1.5) |  | 50 mg | 8 weeks | Early-phase |  | 58.2 (11.9) | 63.7 (10.5) |
| **Tajik-Esmaeeli et al., 2017** | Statin (Simvastatin) | 33 / 33 |  | 31/2 / 28/5 |  | 43.18 (8.89) / 44.64 (9.11) |  | 20.55 (10.79) / 19.60 (10.23) |  | 40 mg | 8 weeks | n/a |  | 47.09 (7.60) | 48.18 (7.70) |
| **Vincenzi et al., 2014** | Statin (Pravastatin) | 30 / 30 |  | 22/8 / 16/14 |  | 42.57 (11) / 44.53 (12.55) |  | 20.89 (13.13) / 22.03 (6.74) |  | 40 mg | 12 weeks | n/a |  | 75.16 (21.87) | 79.93 (18.53) |
| **Hong et al., 2011** | Varenicline | 32 / 32 |  | 20/12 / 22/10 |  | 44.03 / 41.57 |  | n/a |  | 1 mg | 8 weeks | n/a |  | BPRS: 34.13 (1.44) | BPRS: 34.69 (1.52) |
| **Smith et al., 2016** | Varenicline | 42 / 45 |  | 35/7 / 39/6 |  | 46.6 (8.9) / 43.6 (10.6) |  | n/a |  | 2 mg | 8 weeks | Chronic |  | 56.2 (14.9) | 58.8 (15.7) |
| **Chengappa et al., 2018** | Withania somnifera extract | 34 / 34 |  | 21/13 / 14/20 |  | 45.18 (12.90) / 47.38 (11.37) |  | 20.85 (12.26) / 23.38 (11.61) |  | 1000 mg | 12 weeks | Chronic |  | 69.88 (8) | 69.48 (8.45) |
|  |  |  |  |  |  |  |  |  |  |  |  |  |  |  |  |
| α-LA, alpha-lipoic acid ; ALA, α-linolenic acid; BPRS, Brief Psychiatric Rating Scale; Chronic, chronic schizophrenia with an illness duration > 5 years; EPA, eicosapentaenoic acid; DHA, docosahexaenoic acid; Early-phase, early-phase schizophrenia with an illness duration ≤ 5 years; FEP, first-episode psychosis; OA, oleic acid; n/a, not applicable; PANSS, Positive and Negative Syndrome Scale; SD, standard deviation. *Different treatment doses or treatment types were applied within the same study | | | | | | | | | | | | | | | |
|  |  |  |  |  |  |  |  |  |  |  |  |  |  |  |  |
|  |  |  |  |  |  |  |  |  |  |  |  |  |  |  |  |
|  |  |  |  |  |  |  |  |  |  |  |  |  |  |  |  |

| **Supplementary Table 2.** Results of the Cochrane risk of bias tool for randomized trials | | | | | | | |
| --- | --- | --- | --- | --- | --- | --- | --- |
| **Study** | **Random sequence generation** | **Allocation concealment** | **Blinding of participants and personnel** | **Blinding of outcome assessment** | **Incomplete outcome data** | **Selective reporting** | **Total** |
| Akhondzadeh et al., 2003 | **+** | **+** | **+** | **+** | **+** | **+** | **4** |
| Akhondzadeh et al., 2007 | **+** | **+** | **+** | **+** | **+** | **+** | **4** |
| Berger et al., 2007 | **+** | **+** | **+** | **+** | **+** | **+** | **4** |
| Berk et al., 2008 | **+** | **+** | **+** | **+** | **+** | **+** | **4** |
| Boskovic et al., 2016 | **?** | **?** | **+** | **+** | **+** | **+** | **3** |
| Breier et al., 2018 | **?** | **+** | **+** | **+** | **+** | **+** | **3** |
| Chaudhry et al., 2012 | **+** | **+** | **+** | **+** | **+** | **+** | **4** |
| Chaves et al., 2015 | **+** | **+** | **+** | **+** | **+** | **+** | **4** |
| Chengappa et al., 2018 | **+** | **+** | **+** | **+** | **+** | **+** | **4** |
| Deakin et al., 2018 | **+** | **+** | **+** | **+** | **+** | **+** | **4** |
| Emsley et al., 2002 | **?** | **?** | **+** | **+** | **+** | **+** | **3** |
| Emsley et al., 2006 | **+** | **+** | **+** | **+** | **+** | **+** | **4** |
| Emsley et al., 2014 | **+** | **+** | **+** | **+** | **+** | **+** | **4** |
| Farokhnia et al., 2013 | **+** | **+** | **+** | **+** | **+** | **+** | **4** |
| Fenton et al., 2001 | **?** | **+** | **+** | **+** | **+** | **+** | **3** |
| Ghafari et al., 2013 | **+** | **?** | **+** | **+** | **+** | **+** | **3** |
| Ghanizadeh et al., 2014 | **+** | **+** | **+** | **+** | **+** | **+** | **4** |
| Hong et al., 2011 | **?** | **?** | **+** | **+** | **+** | **+** | **3** |
| Iranpour et al., 2016 | **+** | **+** | **+** | **+** | **+** | **+** | **4** |
| Jamilian et al., 2017 | **?** | **+** | **+** | **+** | **+** | **+** | **3** |
| Javitt et al. 2012 | **?** | **?** | **+** | **+** | **+** | **+** | **3** |
| Kelly et al., 2015 | **+** | **+** | **+** | **+** | **+** | **+** | **4** |
| Khodaie-Ardakani et al., 2014 | **+** | **+** | **+** | **+** | **+** | **+** | **4** |
| Khodaie-Ardakani et al., 2015 | **+** | **+** | **+** | **+** | **+** | **+** | **4** |
| Kianimehr et al., 2014 | **?** | **?** | **+** | **+** | **+** | **+** | **3** |
| Kulkarni et al., 2001 | **?** | **+** | **+** | **+** | **+** | **+** | **3** |
| Kulkarni et al., 2008 | **+** | **+** | **+** | **+** | **+** | **+** | **4** |
| Kulkarni et al., 2011 | **?** | **?** | **+** | **+** | **+** | **+** | **3** |
| Kulkarni et al., 2016 | **+** | **+** | **+** | **+** | **+** | **+** | **4** |
| Laan et al., 201 | **-** | **-** | **+** | **+** | **+** | **+** | **3** |
| Lee et al., 2015 | **?** | **?** | **+** | **+** | **+** | **+** | **3** |
| Lerner et al., 2013 | **+** | **+** | **+** | **+** | **+** | **+** | **4** |
| Levkovitz et al., 2010 | **?** | **?** | **+** | **+** | **+** | **+** | **3** |
| Liu et al., 2014 | **?** | **+** | **+** | **+** | **+** | **+** | **3** |
| Louzã et al., 2004 | **?** | **?** | **+** | **+** | **+** | **+** | **3** |
| Modabbernia et al., 2014 | **+** | **+** | **+** | **+** | **+** | **+** | **4** |
| Müller et al., 2002 | **?** | **+** | **+** | **+** | **+** | **+** | **3** |
| Müller et al., 2010 | **?** | **+** | **+** | **+** | **+** | **+** | **3** |
| Noorbala et al., 1999 | **?** | **?** | **+** | **+** | **+** | **+** | **3** |
| Pawełczyk et al., 2016 | **+** | **-** | **+** | **+** | **+** | **+** | **3** |
| Peet and Horobin et al., 2002 | **+** | **+** | **+** | **+** | **+** | **+** | **4** |
| Peet et al., 2001 | **+** | **+** | **+** | **+** | **+** | **+** | **4** |
| Rapaport et al., 2005 | **?** | **?** | **+** | **+** | **+** | **+** | **3** |
| Rappard & Müller 2004 [abstract] | **n/a** | **n/a** | **n/a** | **n/a** | **n/a** | **n/a** | **n/a** |
| Ritsner et al., 2014 | **+** | **?** | **+** | **+** | **+** | **+** | **3** |
| Sepehrmanesh et al., 2018 | **+** | **+** | **+** | **+** | **+** | **+** | **4** |
| Smith et al., 2016 | **+** | **+** | **+** | **+** | **+** | **+** | **4** |
| Tajik-Esmaeeli et al., 2017 | **+** | **+** | **+** | **+** | **+** | **+** | **4** |
| Usall et al., 2016 | **+** | **+** | **+** | **+** | **+** | **+** | **4** |
| Vincenzi et al., 2014 | **?** | **?** | **+** | **+** | **+** | **+** | **3** |
| Weiser et al., 2012 [abstract] | **n/a** | **n/a** | **n/a** | **n/a** | **n/a** | **n/a** | **n/a** |
| Weiser et al., 2017 | **?** | **?** | **+** | **+** | **+** | **+** | **3** |
| Weiser et al., 2019 | **+** | **?** | **+** | **+** | **+** | **+** | **3** |
| Zhang et al., 2015 (Chinese article) | **?** | **?** | **+** | **+** | **+** | **+** | **3** |
| Zhang et al., 2018 | **+** | **+** | **+** | **+** | **+** | **+** | **4** |
|  |  |  |  |  |  |  |  |
| **Key** |  |  |  |  |  |  |  |
| **+** | **Low risk of bias** | |  |  |  |  |  |
| **-** | **High risk of bias** | |  |  |  |  |  |
| **?** | **Unclear risk of bias** | |  |  |  |  |  |

| **Supplementary Table 3.** Subgroup analysis and moderators | | | | | |
| --- | --- | --- | --- | --- | --- |
|  |  |  |  |  |  |
| **Anti-inflammatory agent** | **No. analyses** | **Hedges' *g*** | **95% lower limit** | **95% upper limit** | **P value** |
|  |  |  |  |  |  |
| **Celecoxib** |  |  |  |  |  |
| QASS (reference 4) | 4 | -0.663 | -1.720 | 0.394 | 0.219 |
|  |  |  |  |  |  |
| Illness stage |  |  |  |  |  |
| FEP | 1 | 0.531 | -0.024 | 1.087 | 0.061 |
| Chronic | 4 | 0.051 | -0.899 | 1.001 | 0.916 |
|  |  |  |  |  |  |
| **EPA and DHA Fatty Acids** |  |  |  |  |  |
| QASS (reference 4) | 12 | 0.059 | -0.377 | 0.496 | 0.790 |
| Illness duration | 12 | 0.004 | -0.026 | 0.034 | 0.783 |
| Treatment duration | 14 | 0.007 | -0.023 | 0.036 | 0.658 |
| Treatment dose | 14 | 0.000 | -0.000 | 0.000 | 0.074 |
| Baseline symptom severity | 13 | 0.001 | -0.011 | 0.013 | 0.889 |
|  |  |  |  |  |  |
| Illness stage |  |  |  |  |  |
| FEP | 2 | 0.312 | -0.018 | 0.642 | 0.064 |
| Early-phase | 1 | 0.569 | -0.136 | 1.275 | 0.114 |
| Chronic | 6 | 0.155 | -0.124 | 0.434 | 0.276 |
|  |  |  |  |  |  |
| **Estrogens** |  |  |  |  |  |
| QASS (reference 4) | 12 | -0.259 | -1.092 | 0.575 | 0.543 |
| Illness duration | 7 | -0.011 | -0.078 | 0.056 | 0.753 |
| Treatment duration | 12 | -0.024 | -0.092 | 0.043 | 0.479 |
| Treatment dose | 9 | -0.006 | -0.016 | 0.004 | 0.216 |
| Baseline symptom severity | 9 | 0.001 | -0.024 | 0.026 | 0.943 |
|  |  |  |  |  |  |
| **Minocycline** |  |  |  |  |  |
| QASS (reference 4) | 12 | -0.364 | -1.045 | 0.318 | 0.296 |
| Illness duration | 9 | 0.014 | -0.027 | 0.056 | 0.495 |
| Treatment duration | 12 | -0.012 | -0.029 | 0.006 | 0.198 |
| Treatment dose | 12 | -0.003 | -0.009 | 0.004 | 0.394 |
| Baseline symptom severity | 10 | -0.013 | -0.037 | 0.012 | 0.305 |
| Illness stage |  |  |  |  |  |
| Early-phase | 7 | 0.381 | -0.016 | 0.778 | 0.060 |
| Chronic | 3 | 0.617 | -0.267 | 1.501 | 0.171 |
|  |  |  |  |  |  |
| ***N*-acetylcysteine** |  |  |  |  |  |
| QASS (reference 4) | 5 | 0.359 | -0.404 | 1.123 | 0.356 |
| Illness stage |  |  |  |  |  |
| FEP | 1 | 1.417 | 1.020 | 1.813 | **0.000** |
| Early-phase | 1 | 0.977 | 0.448 | 1.506 | **0.000** |
| Chronic | 1 | 0.441 | 0.107 | 0.774 | **0.010** |
|  |  |  |  |  |  |
| Chronic, chronic schizophrenia with an illness duration > 5 years; Early-phase, early-phase schizophrenia with an illness duration ≤ 5 years; EPA, eicosapentaenoic acid; DHA, docosahexaenoic acid; FEP, first-episode psychosis; QASS, quality assessment score. | | | | | |
|  |  |  |  |  |  |
|  |  |  |  |  |  |

| **Supplementary Table 4.** Effects of anti-inflammatory agents on cognitive domains | | | | | | | | | | | | | | | | | |
| --- | --- | --- | --- | --- | --- | --- | --- | --- | --- | --- | --- | --- | --- | --- | --- | --- | --- |
|  |  |  |  |  |  |  |  |  |  |  |  |  |  |  |  |  |  |
|  | **Aspirin** | **Davunetide** | **Fatty acids** | | **Estrogens** | | **Minocycline** | | | | | | **NAC** | | **Statin** | **Varenicline** | |
| ***Study*** | *Laan et al., 2010* | *Javitt et al., 2012* | *Fenton et al., 2001* | *Emsley et al., 2014* | *Kulkarni et al., 2016* | *Weiser et al., 2017* | *Chaudhry et al., 2012* | *Deakin et al., 2018* | *Levkotvitz et al., 2010* | *Liu et al., 2014* | *Weiser et al., 2019* | *Kelly et al., 2015* | *Breier et al., 2018* | *Sepehrmanesh et al., 2018* | *Vincenzi et al., 2014* | *Hong et al. 2011* | *Smith et al., 2016* |
| **Attention / vigilance** | **-** | **-** | **-** | **-** | **-** | **-** | **-** | n/a | **-** | **+** | **-** | **-** | **-** | n/a | **-** | **-** | **-** |
| **Communication** | n/a | **-** | n/a | n/a | n/a | n/a | n/a | n/a | n/a | n/a | n/a | n/a | n/a | n/a | n/a | n/a | n/a |
| **Comprehension/ Planning** | n/a | **-** | n/a | n/a | n/a | n/a | n/a | n/a | n/a | n/a | n/a | n/a | n/a | n/a | n/a | n/a | n/a |
| **Executive functions** | n/a | n/a | n/a | n/a | n/a | **-** | **-** | n/a | **+** | **-** | **-** | n/a | **-** | n/a | n/a | n/a | n/a |
| **Financial Skills** | n/a | **-** | n/a | n/a | n/a | n/a | n/a | n/a | n/a | n/a | n/a | n/a | n/a | n/a | n/a | n/a | n/a |
| **Fine locomotor skills** | **-** | n/a | n/a | n/a | n/a | n/a | n/a | n/a | n/a | **-** | n/a | n/a | n/a | n/a | n/a | n/a | n/a |
| **Household Management** | n/a | **-** | n/a | n/a | n/a | n/a | n/a | n/a | n/a | n/a | n/a | n/a | n/a | n/a | n/a | n/a | n/a |
| **IQ** | n/a | n/a | n/a | n/a | n/a | n/a | n/a | **-** | n/a | n/a | n/a | n/a | n/a | n/a | n/a | n/a | n/a |
| **Language** | n/a | n/a | **-** | n/a | **-** | n/a | n/a | n/a | n/a | n/a | n/a | n/a | n/a | n/a | n/a | n/a | n/a |
| **Medication Management** | n/a | **-** | n/a | n/a | n/a | n/a | n/a | n/a | n/a | n/a | n/a | n/a | n/a | n/a | n/a | n/a | n/a |
| **Memory** | **-** | n/a | n/a | n/a | n/a | n/a | n/a | n/a | n/a | n/a | n/a | n/a | n/a | n/a | n/a | n/a | n/a |
| **Memory (delayed memory)** | n/a | n/a | **-** | n/a | **-** | n/a | n/a | n/a | n/a | n/a | n/a | n/a | n/a | n/a | n/a | n/a | n/a |
| **Memory (immediate memory)** | n/a | n/a | **-** | n/a | **-** | n/a | n/a | n/a | n/a | n/a | n/a | n/a | n/a | n/a | n/a | n/a | n/a |
| **Memory (verbal memory)** | n/a | n/a | n/a | n/a | n/a | **-** | n/a | n/a | n/a | n/a | **-** | n/a | **-** | n/a | n/a | n/a | n/a |
| **Memory (visuospatial memory)** | n/a | n/a | n/a | n/a | n/a | n/a | **-** | n/a | **+** | n/a | n/a | n/a | n/a | n/a | n/a | n/a | n/a |
| **Memory (working memory)** | n/a | **+ (5 mg only)** | n/a | **-** | n/a | **-** | n/a | n/a | n/a | **-** | **-** | **-** | **-** | n/a | **-** | n/a | **-** |
| **Motor speed** | n/a | n/a | n/a | n/a | n/a | **-** | n/a | n/a | n/a | n/a | **-** | n/a | **-** | n/a | n/a | n/a | n/a |
| **Problem solving** | n/a | n/a | n/a | **-** | n/a | n/a | n/a | n/a | n/a | **-** | n/a | **-** | n/a | n/a | **-** | n/a | **-** |
| **Processing speed** | n/a | **-** | n/a | **-** | n/a | n/a | n/a | **-** | n/a | **-** | n/a | **-** | n/a | n/a | n/a | **-** | **-** |
| **Psychomotor skills** | **-** | n/a | n/a | n/a | n/a | n/a | n/a | n/a | n/a | **-** | n/a | n/a | n/a | n/a | n/a | n/a | n/a |
| **Psychomotor speed** | n/a | n/a | n/a | n/a | n/a | n/a | **-** | n/a | **-** | n/a | n/a | n/a | n/a | n/a | n/a | n/a | n/a |
| **Reasoning / problem-solving** | n/a | **-** | n/a | n/a | n/a | n/a | n/a | n/a | n/a | n/a | n/a | n/a | n/a | n/a | n/a | n/a | n/a |
| **Social cognition** | n/a | **-** | n/a | **-** | n/a | n/a | n/a | n/a | n/a | n/a | n/a | **-** | n/a | n/a | **-** | n/a | **-** |
| **Transportation** | n/a | **-** | n/a | n/a | n/a | n/a | n/a | n/a | n/a | n/a | n/a | n/a | n/a | n/a | n/a | n/a | n/a |
| **Verbal fluency** | n/a | n/a | n/a | n/a | n/a | **-** | n/a | n/a | n/a | **-** | **-** | n/a | **-** | n/a | n/a | n/a | n/a |
| **Verbal learning** | n/a | **+ (5 mg only)** | n/a | **-** | n/a | n/a | n/a | n/a | n/a | **-** | n/a | **-** | n/a | n/a | **-** | n/a | **-** |
| **Visual learning** | n/a | **-** | n/a | **-** | n/a | n/a | n/a | n/a | n/a | **-** | n/a | **-** | n/a | n/a | **-** | n/a | **-** |
| **Visuospatial / constructional** | n/a | n/a | **-** | n/a | **-** | n/a | n/a | n/a | n/a | **-** | n/a | n/a | n/a | n/a | n/a | n/a | n/a |
|  |  |  |  |  |  |  |  |  |  |  |  |  |  |  |  |  |  |
| **Cognitive tools** | RAVLT; HQ-CPT; PPT; TMT | UPSA; MCCB | RBANS | MCCB | RBANS | BACS | CANTAB | Wechsler Adult Intelligence Scale III | CANTAB | MCCB; WCST | BACS | MATRICS/MCCB | BACS | MMSE; DSFBT; DSST; SCWT | MATRICS; UPSA-B | MCCB | MCCB |
|  |  |  |  |  |  |  |  |  |  |  |  |  |  |  |  |  |  |

|  |  |  |  |  |  |  |  |  |  |  |  |
| --- | --- | --- | --- | --- | --- | --- | --- | --- | --- | --- | --- |
| ***Study*** | *Sepehrmanesh et al., 2018* |  |  |  |  |  |  |  |  |  |  |
| **MMSE (visuospatial / language / attention / concentration / memory recall / orientation)*** | **+** |  |  |  |  |  |  |  |  |  |  |
| **The Digit Span test, Backward (Verbal ability / Memory short term)*** | **+** |  |  |  |  |  |  |  |  |  |  |
| **The Digit Span test, Forward (Verbal ability / Memory short term)*** | **+** |  | BACS, Brief Assessment of Cognition in Schizophrenia; CANTAB, Cambridge Neuropsychological Test Automated Battery; DGSBT, Digit Span Forward and Backward Test; DSST, Digit Symbol Substitution Test; HQ-CPT, HQ Continuous Performance Test; IQ, Intelligence Quotient; MCCB, MATRICS (Measurement and Treatment Research to Improve Cognition in Schizophrenia) Consensus Cognitive Battery; MMSE, Mini-Mental State Examination; n/a, not available; NAC, *N*-acetylcysteine; PPT, Purdue Pegboard Test; RAVLT, Rey Auditory Verbal Learning Test; RBANS, Repeatable Battery for the Assessment of Neuropsychological Status; SCWT, Stroop Color-Word Test; TMT, Trail Making Test; UPSA, University of California, San Diego Performance-Based Skills Assessment; Brief Wisconsin Card Sorting Test, WCST. ***** Cognitive domains were not subdivided; **+**, positive significant effects on improving cognition (p-value < 0.05); **-**, no significant effects on improving cognition. | | | | | | | | |
| **The Digit Symbol Substitution Test (brain damage / dementia / age / depression)*** | **+** |  |  |  |  |  |  |  |  |  |  |
| **The stroop test (processing speed / executive functions / working memory / cognitive development)*** | **+** |  |  |  |  |  |  |  |  |  |  |

| **Supplementary Table 5.** Meta-analysis of RCTs with aspirin, estrogens, minocycline, and *N*-acetylcysteine in schizophrenia: efficacy signal | | | |
| --- | --- | --- | --- |
| Anti-inflammatory component | Positive symptoms | Negative symptoms | Cognition* |
|  |  |  |  |
| Aspirin | ES = 0.23; *p* = 0.336 | ES = 0.28; *p* = 0.243 | - |
| Estrogens | **ES = 0.48; *p* = 0.004** | **ES = 0.45; *p* = 0.006** | - |
| Minocycline | ES = 0.14; *p* = 0.094 | **ES = 0.50; *p* = 0.003** | **+/-** |
| *N-*acetylcysteine | ES = 0.21; *p* = 0.124 | **ES = 0.75; *p* = 0.009** | **+/-** |
|  |  |  |  |
| *Heterogeneity of the used cognitive tests across the studies was too great to make a quantitative review of these effects; -, probably no effects; +/-, probably some beneficial effects. | | | |
| ES, mean weighted effect size; *p*, *p*-value; RCTs, randomized controlled trials. | | | |

**Supplementary Figure 1.** Funnel Plot for Estrogen Analysis

**Supplementary Figure 2.** Funnel Plot for Fatty Acids Analysis

**Supplementary Figure 3.** Funnel Plot for Minocycline Analysis

**Forest Plots Showing Effect Size Estimates for the Total Symptom Scores**

**Supplementary Figure 4.** Forest Plot Showing Effect Sizes for Aspirin Augmentation

**Supplementary Figure 5.** Forest Plot Showing Effect Size for Bexarotene Augmentation

**Supplementary Figure 6.** Forest Plot Showing Effect Sizes for Celecoxib Augmentation

**Supplementary Figure 7.** Forest Plot Showing Effect Sizes for Davunetide Augmentation

**Supplementary Figure 8.** Forest Plot Showing Effect Size for Dextromethorphan Augmentation

**Supplementary Figure 9a.** Forest Plot Showing Effect Sizes for Fatty Acids Augmentation

**Supplementary Figure 9b.** Forest Plot Showing Effect Sizes for Fatty Acids Augmentation

**Supplementary Figure 9c.** Forest Plot Showing Effect Sizes for Fatty Acids Augmentation

**Supplementary Figure 9d.** Forest Plot Showing Effect Sizes for Fatty Acids Augmentation

**Supplementary Figure 10a.** Forest Plot Showing Effect Sizes for Estrogen Augmentation

**Supplementary Figure 10b.** Forest Plot Showing Effect Sizes for Estrogen Augmentation

**Supplementary Figure 10c.** Forest Plot Showing Effect Sizes for Estrogen Augmentation

**Supplementary Figure 11.** Forest Plot Showing Effect Size for Melatonin Augmentation

**Supplementary Figure 12a.** Forest Plot Showing Effect Sizes for Minocycline Augmentation

**Supplementary Figure 12b.** Forest Plot Showing Effect Sizes for Minocycline Augmentation

**Supplementary Figure 13.** Forest Plot Showing Effect Sizes for *N*-acetylcysteine (NAC) Augmentation

**Supplementary Figure 14.** Forest Plot Showing Effect Size for Pioglitazone Augmentation

**Supplementary Figure 15.** Forest Plot Showing Effect Size for Piracetam Augmentation

**Supplementary Figure 16.** Forest Plot Showing Effect Size for Pregnenolone Augmentation

**Supplementary Figure 17.** Forest Plot Showing Effect Sizes for Statin Augmentation

**Supplementary Figure 18.** Forest Plot Showing Effect Sizes for Varenicline Augmentation

**Supplementary Figure 19.** Forest Plot Showing Effect Size for Withania Somnifera Extract (WSE) Augmentation

**Forest Plots Showing Effect Size Estimates for the Positive Symptom Scores**

**Supplementary Figure 20.** Forest Plot Showing Effect Size for Aspirin Augmentation

**Supplementary Figure 21.** Forest Plot Showing Effect Size for Bexarotene Augmentation

**Supplementary Figure 22.** Forest Plot Showing Effect Sizes for Celecoxib Augmentation

**Supplementary Figure 23.** Forest Plot Showing Effect Sizes for Estrogen Augmentation

**Supplementary Figure 24.** Forest Plot Showing Effect Sizes for Fatty Acids Augmentation

**Supplementary Figure 25.** Forest Plot Showing Effect Size for Melatonin Augmentation

**Supplementary Figure 26.** Forest Plot Showing Effect Sizes for Minocycline Augmentation

**Supplementary Figure 27.** Forest Plot Showing Effect Sizes for NAC Augmentation

**Supplementary Figure 28.** Forest Plot Showing Effect Size for Pioglitazone Augmentation

**Supplementary Figure 29.** Forest Plot Showing Effect Size for Piracetam Augmentation

**Supplementary Figure 30.** Forest Plot Showing Effect Size for Pregnenolone Augmentation

**Supplementary Figure 31.** Forest Plot Showing Effect Sizes for Statin Augmentation

**Supplementary Figure 32.** Forest Plot Showing Effect Size for Varenicline Augmentation

**Supplementary Figure 33.** Forest Plot Showing Effect Size for WSE Augmentation

**Forest Plots Showing Effect Size Estimates for the Negative Symptom Scores**

**Supplementary Figure 34.** Forest Plot Showing Effect Size for Aspirin Augmentation

**Supplementary Figure 35.** Forest Plot Showing Effect Size for Bexarotene Augmentation


**Supplementary Figure 36.** Forest Plot Showing Effect Sizes for Celecoxib Augmentation

**Supplementary Figure 37.** Forest Plot Showing Effect Sizes for Estrogen Augmentation

**Supplementary Figure 38.** Forest Plot Showing Effect Sizes for Fatty Acids Augmentation

**Supplementary Figure 39.** Forest Plot Showing Effect Size for Melatonin Augmentation

**Supplementary Figure 40.** Forest Plot Showing Effect Sizes for Minocycline Augmentation

**Supplementary Figure 41.** Forest Plot Showing Effect Sizes for NAC Augmentation

**Supplementary Figure 42.** Forest Plot Showing Effect Size for Pioglitazone Augmentation


**Supplementary Figure 43.** Forest Plot Showing Effect Size for Pregnenolone Augmentation

**Supplementary Figure 44.** Forest Plot Showing Effect Sizes for Statin Augmentation

**Supplementary Figure 45.** Forest Plot Showing Effect Size for Varenicline Augmentation


**Supplementary Figure 46.** Forest Plot Showing Effect Size for WSE Augmentation

**References**

**Akhondzadeh S, Nejatisafa AA, Amini H, Mohammadi MR, Larijani B, Kashani L, Raisi F and Kamalipour A** (2003). Adjunctive estrogen treatment in women with chronic schizophrenia: a double-blind, randomized, and placebo-controlled trial. *Progress in Neuro-Psychopharmacology and Biological Psychiatry* **27**, 1007-12.

**Akhondzadeh S, Tabatabaee M, Amini H, Ahmadi Abhari SA, Abbasi SH and Behnam B** (2007). Celecoxib as adjunctive therapy in schizophrenia: a double-blind, randomized and placebo-controlled trial. *Schizophrenia Research* **90**, 179-85.

**Bentsen H, Osnes K, Refsum H, Solberg DK and Bohmer T** (2013). A randomized placebo-controlled trial of an omega-3 fatty acid and vitamins E+C in schizophrenia. *Translational Psychiatry* **3**, e335.

**Berger GE, Proffitt TM, McConchie M, Yuen H, Wood SJ, Amminger GP, Brewer W and McGorry PD** (2007). Ethyl-eicosapentaenoic acid in first-episode psychosis: a randomized, placebo-controlled trial. *Journal of Clinical Psychiatry* **68**, 1867-75.

**Berk M, Copolov D, Dean O, Lu K, Jeavons S, Schapkaitz I, Anderson-Hunt M, Judd F, Katz F, Katz P, Ording-Jespersen S, Little J, Conus P, Cuenod M, Do KQ and Bush AI** (2008). N-acetyl cysteine as a glutathione precursor for schizophrenia--a double-blind, randomized, placebo-controlled trial. *Biological Psychiatry* **64**, 361-8.

**Boskovic M, Vovk T, Koprivsek J, Plesnicar BK and Grabnar I** (2016). Vitamin E and essential polyunsaturated fatty acids supplementation in schizophrenia patients treated with haloperidol. *Nutritional Neuroscience* **19**, 156-61.

**Breier A, Liffick E, Hummer TA, Vohs JL, Yang Z, Mehdiyoun NF, Visco AC, Metzler E, Zhang Y and Francis MM** (2018). Effects of 12-month, double-blind N-acetyl cysteine on symptoms, cognition and brain morphology in early phase schizophrenia spectrum disorders. *Schizophrenia Research* **199**, 395-402.

**Chaudhry IB, Hallak J, Husain N, Minhas F, Stirling J, Richardson P, Dursun S, Dunn G and Deakin B** (2012). Minocycline benefits negative symptoms in early schizophrenia: a randomised double-blind placebo-controlled clinical trial in patients on standard treatment. *Journal of Psychopharmacology* **26**, 1185-93.

**Chaves C, Marque CR, Maia-de-Oliveira JP, Wichert-Ana L, Ferrari TB, Santos AC, Araujo D, Machado-de-Sousa JP, Bressan RA, Elkis H, Crippa JA, Guimaraes FS, Zuardi AW, Baker GB, Dursun SM and Hallak JE** (2015). Effects of minocycline add-on treatment on brain morphometry and cerebral perfusion in recent-onset schizophrenia. *Schizophrenia Research* **161**, 439-45.

**Chengappa KNR, Brar JS, Gannon JM and Schlicht PJ** (2018). Adjunctive Use of a Standardized Extract of Withania somnifera (Ashwagandha) to Treat Symptom Exacerbation in Schizophrenia: A Randomized, Double-Blind, Placebo-Controlled Study. *Journal of Clinical Psychiatry* **79**.

**Deakin B, Suckling J, Barnes TRE, Byrne K, Chaudhry IB, Dazzan P, Drake RJ, Giordano A, Husain N, Jones PB, Joyce E, Knox E, Krynicki C, Lawrie SM, Lewis S, Lisiecka-Ford DM, Nikkheslat N, Pariante CM, Smallman R, Watson A, Williams SCR, Upthegrove R and Dunn G** (2018). The benefit of minocycline on negative symptoms of schizophrenia in patients with recent-onset psychosis (BeneMin): a randomised, double-blind, placebo-controlled trial. *Lancet Psychiatry* **5**, 885-894.

**Emsley R, Chiliza B, Asmal L, du Plessis S, Phahladira L, van Niekerk E, van Rensburg SJ and Harvey BH** (2014). A randomized, controlled trial of omega-3 fatty acids plus an antioxidant for relapse prevention after antipsychotic discontinuation in first-episode schizophrenia. *Schizophrenia Research* **158**, 230-5.

**Emsley R, Myburgh C, Oosthuizen P and van Rensburg SJ** (2002). Randomized, placebo-controlled study of ethyl-eicosapentaenoic acid as supplemental treatment in schizophrenia. *American Journal of Psychiatry* **159**, 1596-8.

**Emsley R, Niehaus DJ, Koen L, Oosthuizen PP, Turner HJ, Carey P, van Rensburg SJ, Maritz JS and Murck H** (2006). The effects of eicosapentaenoic acid in tardive dyskinesia: a randomized, placebo-controlled trial. *Schizophrenia Research* **84**, 112-20.

**Farokhnia M, Azarkolah A, Adinehfar F, Khodaie-Ardakani MR, Hosseini SM, Yekehtaz H, Tabrizi M, Rezaei F, Salehi B, Sadeghi SM, Moghadam M, Gharibi F, Mirshafiee O and Akhondzadeh S** (2013). N-acetylcysteine as an adjunct to risperidone for treatment of negative symptoms in patients with chronic schizophrenia: a randomized, double-blind, placebo-controlled study. *Clinical Neuropharmacology* **36**, 185-92.

**Fenton WS, Dickerson F, Boronow J, Hibbeln JR and Knable M** (2001). A placebo-controlled trial of omega-3 fatty acid (ethyl eicosapentaenoic acid) supplementation for residual symptoms and cognitive impairment in schizophrenia. *American Journal of Psychiatry* **158**, 2071-4.

**Ghafari E, Fararouie M, Shirazi HG, Farhangfar A, Ghaderi F and Mohammadi A** (2013). Combination of estrogen and antipsychotics in the treatment of women with chronic schizophrenia: a double-blind, randomized, placebo-controlled clinical trial. *Clinical Schizophrenia & Related Psychoses* **6**, 172-6.

**Ghanizadeh A, Dehbozorgi S, OmraniSigaroodi M and Rezaei Z** (2014). Minocycline as add-on treatment decreases the negative symptoms of schizophrenia; a randomized placebo-controlled clinical trial. *Recent Patents on Inflammation & Allergy Drug Discovery* **8**, 211-5.

**Hong LE, Thaker GK, McMahon RP, Summerfelt A, Rachbeisel J, Fuller RL, Wonodi I, Buchanan RW, Myers C, Heishman SJ, Yang J and Nye A** (2011). Effects of moderate-dose treatment with varenicline on neurobiological and cognitive biomarkers in smokers and nonsmokers with schizophrenia or schizoaffective disorder. *Archives of General Psychiatry* **68**, 1195-206.

**Howes OD, McCutcheon R, Agid O, de Bartolomeis A, van Beveren NJ, Birnbaum ML, Bloomfield MA, Bressan RA, Buchanan RW, Carpenter WT, Castle DJ, Citrome L, Daskalakis ZJ, Davidson M, Drake RJ, Dursun S, Ebdrup BH, Elkis H, Falkai P, Fleischacker WW, Gadelha A, Gaughran F, Glenthoj BY, Graff-Guerrero A, Hallak JE, Honer WG, Kennedy J, Kinon BJ, Lawrie SM, Lee J, Leweke FM, MacCabe JH, McNabb CB, Meltzer H, Moller HJ, Nakajima S, Pantelis C, Reis Marques T, Remington G, Rossell SL, Russell BR, Siu CO, Suzuki T, Sommer IE, Taylor D, Thomas N, Ucok A, Umbricht D, Walters JT, Kane J and Correll CU** (2017). Treatment-Resistant Schizophrenia: Treatment Response and Resistance in Psychosis (TRRIP) Working Group Consensus Guidelines on Diagnosis and Terminology. *American Journal of Psychiatry* **174**, 216-229.

**Iranpour N, Zandifar A, Farokhnia M, Goguol A, Yekehtaz H, Khodaie-Ardakani MR, Salehi B, Esalatmanesh S, Zeionoddini A, Mohammadinejad P, Zeinoddini A and Akhondzadeh S** (2016). The effects of pioglitazone adjuvant therapy on negative symptoms of patients with chronic schizophrenia: a double-blind and placebo-controlled trial. *Human Psychopharmacology* **31**, 103-12.

**Jamilian H, Solhi H and Jamilian M** (2014). Randomized, placebo-controlled clinical trial of omega-3 as supplemental treatment in schizophrenia. *Global Journal of Health Science* **6**, 103-8.

**Javitt DC, Buchanan RW, Keefe RS, Kern R, McMahon RP, Green MF, Lieberman J, Goff DC, Csernansky JG, McEvoy JP, Jarskog F, Seidman LJ, Gold JM, Kimhy D, Nolan KS, Barch DS, Ball MP, Robinson J and Marder SR** (2012). Effect of the neuroprotective peptide davunetide (AL-108) on cognition and functional capacity in schizophrenia. *Schizophrenia Research* **136**, 25-31.

**Kelly DL, Sullivan KM, McEvoy JP, McMahon RP, Wehring HJ, Gold JM, Liu F, Warfel D, Vyas G, Richardson CM, Fischer BA, Keller WR, Koola MM, Feldman SM, Russ JC, Keefe RS, Osing J, Hubzin L, August S, Walker TM and Buchanan RW** (2015). Adjunctive Minocycline in Clozapine-Treated Schizophrenia Patients With Persistent Symptoms. *Journal of Clinical Psychopharmacology* **35**, 374-81.

**Khodaie-Ardakani MR, Khosravi M, Zarinfard R, Nejati S, Mohsenian A, Tabrizi M and Akhondzadeh S** (2015). A Placebo-Controlled Study of Raloxifene Added to Risperidone in Men with Chronic Schizophrenia. *Acta Medica Iranica* **53**, 337-45.

**Khodaie-Ardakani MR, Mirshafiee O, Farokhnia M, Tajdini M, Hosseini SM, Modabbernia A, Rezaei F, Salehi B, Yekehtaz H, Ashrafi M, Tabrizi M and Akhondzadeh S** (2014). Minocycline add-on to risperidone for treatment of negative symptoms in patients with stable schizophrenia: randomized double-blind placebo-controlled study. *Psychiatry Research* **215**, 540-6.

**Kianimehr G, Fatehi F, Hashempoor S, Khodaei-Ardakani MR, Rezaei F, Nazari A, Kashani L and Akhondzadeh S** (2014). Raloxifene adjunctive therapy for postmenopausal women suffering from chronic schizophrenia: a randomized double-blind and placebo controlled trial. *Daru* **22**, 55.

**Kulkarni J, de Castella A, Fitzgerald PB, Gurvich CT, Bailey M, Bartholomeusz C and Burger H** (2008). Estrogen in severe mental illness: a potential new treatment approach. *Archives of General Psychiatry* **65**, 955-60.

**Kulkarni J, de Castella A, Headey B, Marston N, Sinclair K, Lee S, Gurvich C, Fitzgerald PB and Burger H** (2011). Estrogens and men with schizophrenia: is there a case for adjunctive therapy? *Schizophrenia Research* **125**, 278-83.

**Kulkarni J, Gavrilidis E, Gwini SM, Worsley R, Grigg J, Warren A, Gurvich C, Gilbert H, Berk M and Davis SR** (2016). Effect of Adjunctive Raloxifene Therapy on Severity of Refractory Schizophrenia in Women: A Randomized Clinical Trial. *JAMA Psychiatry* **73**, 947-54.

**Kulkarni J, Riedel A, de Castella AR, Fitzgerald PB, Rolfe TJ, Taffe J and Burger H** (2001). Estrogen - a potential treatment for schizophrenia. *Schizophrenia Research* **48**, 137-44.

**Laan W, Grobbee DE, Selten JP, Heijnen CJ, Kahn RS and Burger H** (2010). Adjuvant aspirin therapy reduces symptoms of schizophrenia spectrum disorders: results from a randomized, double-blind, placebo-controlled trial. *Journal of Clinical Psychiatry* **71**, 520-7.

**Lee SY, Chen SL, Chang YH, Chen PS, Huang SY, Tzeng NS, Wang LJ, Lee IH, Wang TY, Chen KC, Yang YK, Hong JS and Lu RB** (2015). ALDH2 polymorphism, associated with attenuating negative symptoms in patients with schizophrenia treated with add-on dextromethorphan. *Journal of Psychiatric Research* **69**, 50-6.

**Lerner V, Miodownik C, Gibel A, Sirota P, Bush I, Elliot H, Benatov R and Ritsner MS** (2013). The retinoid X receptor agonist bexarotene relieves positive symptoms of schizophrenia: a 6-week, randomized, double-blind, placebo-controlled multicenter trial. *Journal of Clinical Psychiatry* **74**, 1224-32.

**Levkovitz Y, Mendlovich S, Riwkes S, Braw Y, Levkovitch-Verbin H, Gal G, Fennig S, Treves I and Kron S** (2010). A double-blind, randomized study of minocycline for the treatment of negative and cognitive symptoms in early-phase schizophrenia. *Journal of Clinical Psychiatry* **71**, 138-49.

**Liu F, Guo X, Wu R, Ou J, Zheng Y, Zhang B, Xie L, Zhang L, Yang L, Yang S, Yang J, Ruan Y, Zeng Y, Xu X and Zhao J** (2014). Minocycline supplementation for treatment of negative symptoms in early-phase schizophrenia: a double blind, randomized, controlled trial. *Schizophrenia Research* **153**, 169-76.

**Louza MR, Marques AP, Elkis H, Bassitt D, Diegoli M and Gattaz WF** (2004). Conjugated estrogens as adjuvant therapy in the treatment of acute schizophrenia: a double-blind study. *Schizophrenia Research* **66**, 97-100.

**Modabbernia A, Heidari P, Soleimani R, Sobhani A, Roshan ZA, Taslimi S, Ashrafi M and Modabbernia MJ** (2014). Melatonin for prevention of metabolic side-effects of olanzapine in patients with first-episode schizophrenia: randomized double-blind placebo-controlled study. *Journal of Psychiatric Research* **53**, 133-40.

**Muller N, Krause D, Dehning S, Musil R, Schennach-Wolff R, Obermeier M, Moller HJ, Klauss V, Schwarz MJ and Riedel M** (2010). Celecoxib treatment in an early stage of schizophrenia: results of a randomized, double-blind, placebo-controlled trial of celecoxib augmentation of amisulpride treatment. *Schizophrenia Research* **121**, 118-24.

**Muller N, Riedel M, Scheppach C, Brandstatter B, Sokullu S, Krampe K, Ulmschneider M, Engel RR, Moller HJ and Schwarz MJ** (2002). Beneficial antipsychotic effects of celecoxib add-on therapy compared to risperidone alone in schizophrenia. *American Journal of Psychiatry* **159**, 1029-34.

**Noorbala AA, Akhondzadeh S, Davari-Ashtiani R and Amini-Nooshabadi H** (1999). Piracetam in the treatment of schizophrenia: implications for the glutamate hypothesis of schizophrenia. *Journal of Clinical Pharmacy and Therapeutics* **24**, 369-74.

**Pawelczyk T, Grancow-Grabka M, Kotlicka-Antczak M, Trafalska E and Pawelczyk A** (2016). A randomized controlled study of the efficacy of six-month supplementation with concentrated fish oil rich in omega-3 polyunsaturated fatty acids in first episode schizophrenia. *Journal of Psychiatric Research* **73**, 34-44.

**Peet M, Brind J, Ramchand CN, Shah S and Vankar GK** (2001). Two double-blind placebo-controlled pilot studies of eicosapentaenoic acid in the treatment of schizophrenia. *Schizophrenia Research* **49**, 243-51.

**Peet M and Horrobin DF** (2002). A dose-ranging exploratory study of the effects of ethyl-eicosapentaenoate in patients with persistent schizophrenic symptoms. *Journal of Psychiatric Research* **36**, 7-18.

**Rapaport MH, Delrahim KK, Bresee CJ, Maddux RE, Ahmadpour O and Dolnak D** (2005). Celecoxib augmentation of continuously ill patients with schizophrenia. *Biological Psychiatry* **57**, 1594-6.

**Rappard F and Muller N** (2004). Celecoxib add-on does not have beneficial antipsychotic effects over risperidone alone in schizophrenia [abstract]. *Neuropsychopharmacology* **29**.

**Ritsner MS, Bawakny H and Kreinin A** (2014). Pregnenolone treatment reduces severity of negative symptoms in recent-onset schizophrenia: an 8-week, double-blind, randomized add-on two-center trial. *Psychiatry and Clinical Neurosciences* **68**, 432-40.

**Sepehrmanesh Z, Heidary M, Akasheh N, Akbari H and Heidary M** (2018). Therapeutic effect of adjunctive N-acetyl cysteine (NAC) on symptoms of chronic schizophrenia: A double-blind, randomized clinical trial. *Progress in Neuro-Psychopharmacology and Biological Psychiatry* **82**, 289-296.

**Smith RC, Amiaz R, Si TM, Maayan L, Jin H, Boules S, Sershen H, Li C, Ren J, Liu Y, Youseff M, Lajtha A, Guidotti A, Weiser M and Davis JM** (2016). Varenicline Effects on Smoking, Cognition, and Psychiatric Symptoms in Schizophrenia: A Double-Blind Randomized Trial. *PloS One* **11**, e0143490.

**Tajik-Esmaeeli S, Moazen-Zadeh E, Abbasi N, Shariat SV, Rezaei F, Salehi B and Akhondzadeh S** (2017). Simvastatin adjunct therapy for negative symptoms of schizophrenia: a randomized double-blind placebo-controlled trial. *International Clinical Psychopharmacology* **32**, 87-94.

**Usall J, Huerta-Ramos E, Labad J, Cobo J, Nunez C, Creus M, Pares GG, Cuadras D, Franco J, Miquel E, Reyes JC and Roca M** (2016). Raloxifene as an Adjunctive Treatment for Postmenopausal Women With Schizophrenia: A 24-Week Double-Blind, Randomized, Parallel, Placebo-Controlled Trial. *Schizophrenia Bulletin* **42**, 309-17.

**Vincenzi B, Stock S, Borba CP, Cleary SM, Oppenheim CE, Petruzzi LJ, Fan X, Copeland PM, Freudenreich O, Cather C and Henderson DC** (2014). A randomized placebo-controlled pilot study of pravastatin as an adjunctive therapy in schizophrenia patients: effect on inflammation, psychopathology, cognition and lipid metabolism. *Schizophrenia Research* **159**, 395-403.

**Weiser M, Burshtein S and L. F** (2012). A randomized trial administering aspirin, minocycline or pramipexole vs placebo as add-on to antipsychotics in patients with schizophrenia or schizoaffective disorder [abstract]. *Neuropsychopharmacology* **38**.

**Weiser M, Levi L, Burshtein S, Chirita R, Cirjaliu D, Gonen I, Yolken R, Davidson M, Zamora D and Davis JM** (2019). The effect of minocycline on symptoms in schizophrenia: Results from a randomized controlled trial. *Schizophrenia Research* **206**, 325-332.

**Weiser M, Levi L, Burshtein S, Hagin M, Matei VP, Podea D, Miclutia I, Tiugan A, Pacala B, Grecu IG, Noy A, Zamora D and Davis JM** (2017). Raloxifene Plus Antipsychotics Versus Placebo Plus Antipsychotics in Severely Ill Decompensated Postmenopausal Women With Schizophrenia or Schizoaffective Disorder: A Randomized Controlled Trial. *Journal of Clinical Psychiatry* **78**, e758-e765.

**Zhang J-h, Chen B and Jian-rui. L** (2015). Treatment effect of risperidone alone and combined with N-acetly-cysteine for first-episode schizophenic patients [Chinese article]. *Journal of Clinical Psychiatry* **25**.

**Zhang L, Zheng H, Wu R, Zhu F, Kosten TR, Zhang XY and Zhao J** (2018). Minocycline adjunctive treatment to risperidone for negative symptoms in schizophrenia: Association with pro-inflammatory cytokine levels. *Progress in Neuro-Psychopharmacology and Biological Psychiatry* **85**, 69-76.
